# lncRNA SNHG6 regulates EZH2 expression by sponging miR-26a/b and miR-214 in colorectal cancer

**DOI:** 10.1186/s13045-018-0690-5

**Published:** 2019-01-09

**Authors:** Mu Xu, Xiaoxiang Chen, Kang Lin, Kaixuan Zeng, Xiangxiang Liu, Xueni Xu, Bei Pan, Tao Xu, Li Sun, Bangshun He, Yuqin Pan, Huiling Sun, Shukui Wang

**Affiliations:** 1 General Clinical Research Center, Nanjing First Hospital, Nanjing Medical University, No. 68, Changle Road, Nanjing, 210006 China; 2 School of Medicine, Southeast University, Nanjing, 210009 China; 3 Department of Laboratory Medicine, Nanjing First Hospital, Nanjing Medical University, Nanjing, 210006 China; 4 Department of Laboratory Medicine, The Second Affiliated Hospital of Nanjing Medical University, Nanjing, 210011 China

**Keywords:** SNHG6, EZH2, microRNA, Colorectal cancer, ceRNA

## Abstract

**Background:**

Abnormal expression of long non-coding RNAs (lncRNAs) has been found in almost all human tumors, providing numerous potential diagnostic biomarkers, prognostic biomarkers, and therapeutic targets.

**Methods:**

We analyzed RNA sequencing data to explore abnormally expressed lncRNAs in colorectal cancer (CRC). The functions of small nucleolar RNA host gene 6 (SNHG6) were investigated through in vitro and in vivo assays (CCK-8 assay, colony formation assay, flow cytometry assay, EdU assay, wound healing assay, transwell assay, and xenograft model). The mechanism of action of SNHG6 was explored through bioinformatics, RNA fluorescence in situ hybridization, luciferase reporter assay, RNA pull-down assay, chromatin immunoprecipitation assay, and RNA immunoprecipitation assay.

**Results:**

We identified aberrantly expressed lncRNAs in CRC. We found that elevated SNHG6 expression was associated with poor prognosis and CRC progression. We also demonstrated that the high SNHG6 expression was partly due to DNA copy number gains and SP1 induction. Functional studies showed that SNHG6 promoted CRC cell growth, migration, and invasion both in vitro and in vivo*.* Mechanistically, we found that SNHG6 expressed predominantly in the cytoplasm. SNHG6 could interact with miR-26a, miR-26b, and miR-214 and regulate their common target EZH2.

**Conclusions:**

Our study elucidated that SNHG6 acted as an oncogene in CRC, which might serve as a novel target for CRC diagnosis and therapy.

**Electronic supplementary material:**

The online version of this article (10.1186/s13045-018-0690-5) contains supplementary material, which is available to authorized users.

## Background

Ranking as the third most commonly diagnosed cancer, colorectal cancer (CRC) is the fourth leading cause of cancer-related death worldwide [1]. Many CRC patients are diagnosed at advanced stages owing to the lack of incipient symptoms and limitations in timely screening methods [2]. In addition, there is a high frequency of recurrence and metastasis in CRC patients, even among those who undergo surgical resection. These factors contribute to the poor prognosis of CRC [3]. Therefore, an investigation into the deep molecular mechanisms of colorectal cancer tumorigenesis and progression is urgently needed to improve early diagnosis and treatment.

Recently, advances in whole-genome sequencing technology have revolutionized our understanding of the human genome. Evidence reveals that more than 90% of the human genome is actively transcribed, but only 2% of the transcripts are responsible for encoding proteins, and most human genome transcripts are non-coding RNAs [4]. Long noncoding RNAs (lncRNAs) are a class of transcripts longer than 200 nucleotides with limited or no protein-coding potential. They have attracted increasing attention, because a growing number of studies have suggested that they were involved in many physiological and pathological processes, such as cell differentiation, cell cycle control, cell apoptosis, cell migration, and cell invasion. lncRNAs play their roles through various mechanisms, such as acting as guides of chromatin-modifying complexes, scaffolds of proteins, decoys of mRNAs, and sponges for miRNAs [5, 6]. Importantly, dysregulated lncRNAs have been frequently observed in various diseases, including cancer, and they are widely reported to participate in cancer cell growth, metastasis, and drug resistance [7, 8].

SNHG6 (small nucleolar RNA host gene 6) is located on chromosome 8q13, a region with frequent copy number amplification in CRC [9–11]. It has been identified as an oncogene in many cancers, such as gastric cancer, glioma, hepatocellular carcinoma, and osteosarcoma [12–17]. Meng et al. reported that high SNHG6 expression predicted poor prognosis in colorectal cancer [18]. However, the biological functions of SNHG6 in CRC remain to be elucidated.

Enhancer of zeste homolog 2 (EZH2) is the enzymatic subunit of polycomb repressor complex 2 (PRC2), which can catalyze the trimethylation of lysine27 of histone 3 (H3K27me3) and result in the transcriptional silencing of target genes. EZH2 is highly expressed and functions as an oncogene in numerous types of cancer. EZH2 expression upregulation is induced by some oncogenic transcription factors and various tumor suppressor miRNAs, such as miR-26a/b, miR-101, and miR-214 [19, 20].

In this study, we assessed aberrantly expressed lncRNAs in CRC and then explored the biological functions of the top upregulated lncRNA SNHG6. We found high SNHG6 expression was associated with CRC progression and predicted poor prognosis. Besides, we demonstrated that high SNHG6 expression was due to DNA copy number gains and SP1 activation. SNHG6 silencing inhibited CRC cell growth, migration, and invasion, while SNHG6 overexpression promoted CRC cell growth, migration, and invasion. We revealed that SNHG6 exerted its oncogenic functions by sponging miR-26a/b and miR-214 and acting as a competing endogenous RNA (ceRNA) for EZH2. Our findings suggest that SNHG6 could be a potential biomarker and treatment target in CRC.

## Methods

### Cell lines

The human colorectal cancer cell lines (HCT-116, HCT-8, SW-480, SW-620, DLD-1, and HT-29) and the human normal colorectal epithelial cell FHC were obtained from American Type Culture Collection. SW-480, SW-620, DLD-1, HT-29, and FHC cells were cultured in Dulbecco’s modified Eagle’s medium (DMEM) with 10% fetal bovine serum. HCT-116 and HCT-8 cells were cultured in RPMI-1640 medium with 10% fetal bovine serum. Cells were maintained in a humidified atmosphere of 5% CO_2_ at 37 °C. All cells were authenticated via Short Tandem Repeat DNA profiling and routinely tested and found negative for mycoplasma infection.

### Tissue samples and clinical data collection

A total of 120 CRC tissue and 80 adjacent nontumor tissue samples were obtained from patients during operations at the Affiliated Nanjing First Hospital of Nanjing Medical University (Nanjing, China). All collected tissue samples were immediately snap frozen in liquid nitrogen and stored at − 80 °C until used. The patient characteristics are listed in Table 1. This study was approved by the ethics committee on Human Research of the Nanjing First Hospital and written informed consents were obtained from all patients.

**Table 1:** The clinic-pathological factors of 120 CRC patients

Characteristics	Number of cases	SNHG6 expression		*P* value^a^
		Low (*n* = 60)	High (*n* = 60)	
Age(year)				
< 60	53	24	29	0.358
≥ 60	67	36	31	
Gender				
Female	58	31	27	0.465
Male	62	29	33	
Tumor invasion depth				
T1–2	83	48	35	*0.010*
T3–4	37	12	25	
Lymph node metastasis				
N0	95	53	42	*0.013*
N1 + N2	25	7	18	
Distant metastasis				
M0	96	55	41	*0.001*
M1 + M2	24	5	19	
TNM stage				
I + II	87	50	37	*0.008*
III + III	33	10	23	

^a^Statistical significant results (in italics)

### Quantitative reverse transcription polymerase chain reaction (qRT-PCR)

Total RNA was isolated from the tissue samples and cells by TRIzol (Invitrogen, USA) according to the manufacturer’s protocol. lncRNA and mRNA detection was performed using a SYBR Green PCR Kit (Takara, Japan) with an ABI 7500 System. GAPDH expression was employed as a control for normalization. MicroRNA detection was performed using a miDETECT A Track Kit (RiboBio, China). The expression of the small nuclear RNA U6 was used as a control for normalization. For SNHG6 copy number detection, genomic DNA was isolated using a Genomic DNA Purification Kit (Promega, USA). QRT-PCR was performed using SYBR Premix Ex Taq (Takara, Japan) with an ABI 7300 System. POLR2A, RPP14, andTBX15 expression levels were employed for normalization. These three loci are housekeeping genes, and their copy numbers are stable across the population. A sample with a mean expression of SNHG6 relative to these reference genes greater than 1.5 is defined as copy number gain. A sample with a mean expression of SNHG6 relative to these reference genes less than 0.6 is defined as copy number loss. Each experiment was repeated at least three times. Primers are listed in Additional file 1: Table S1.

### Plasmid construction and cell transfection

The full-length complementary cDNAs of human SP1 was synthesized and cloned into the expression vector pcDNA3.1 (Invitrogen, China). The full-length complementary cDNA of SNHG6 (or cDNA containing mutations) was synthesized and cloned into the expression vector pLenti-Glll-GMV-GFP-2A-Puro (Applied Biological Materials, Canada). The small hairpin RNA (shRNA) of SNHG6 was synthesized and cloned into the piLenti-shRNA-GFP vector (Applied Biological Materials, Canada). siRNAs were synthesized by GenePharma (China). MicroRNA mimics and inhibitors were synthesized by RiboBio (China). The plasmid vectors and siRNAs were transfected into CRC cells using Lipofectamine 2000 (Invitrogen, USA) according to the manufacturer’s instructions. All siRNA and shRNA sequences are listed in Additional file 1: Table S2.

### Luciferase reporter assay

For the SNHG6 promoter luciferase reporter assay, different fragment sequences containing predicted SP1 binding sites were synthesized and then cloned into the pGL3-basic firefly luciferase reporter (GeneCreat, China). The pRL-TK vector was employed as a control. For the microRNA target gene luciferase reporter assay, target sequences containing the predicted microRNA binding sites (or containing mutations in the predicted microRNA binding sites) were synthesized and inserted into the psiCHECK-2 vector (Promega, USA). Luciferase activity was measured with a Dual Luciferase Assay system (Promega, USA).

### Chromatin immunoprecipitation assay (ChIP)

ChIP assay was performed using the ChIP Assay Kit (Beyotime, China) according to the manual instructions with slight modifications. Briefly, HCT-116 and HCT-8 cells were cross-linked with 1% formaldehyde solution for 10 min at room temperature and quenched with 125 mM glycine. DNA fragments ranging from 200 to 500 bp were obtained via sonication. Then, the lysate was immunoprecipitated with anti-SP1, anti-H3K27me3, or IgG antibody. Immunoprecipitated DNA fragments were analyzed by qRT-PCR. ChIP primers are listed in Additional file 1: Table S1. The antibodies used in ChIP assay are listed in Additional file 1: Table S3.

### Xenograft tumor formation and in vivo metastasis assay

Five-week-old male BALB**/**c nude mice were maintained under specific pathogen-free conditions and manipulated according to the protocols approved by the Animal Care Committee of Nanjing Medical College. Stably transfected HCT-116 cells (5 **×** 10^6^**/**0.2 ml PBS) were implanted into two sides of the same nude mouse in the armpit. Xenografts were examined every 4 days with digital calipers, and tumor volumes were calculated using the following equation: volume = 1**/**2 (length **×** width^2^). Twenty-three days later, the mice were sacrificed, and volumes of tumors were measured. For the in vivo tumor metastasis assay, the indicated cells (3 **×** 10^6^**/**0.2 ml PBS) were injected via the tail vein into nude mice; 60 days later, all mice were euthanized; and the lungs were surgically dissected. The samples were embedded in paraffin for hematoxylin and eosin (HE) staining and immunohistochemistry staining.

### RNA fluorescence in situ hybridization (FISH)

FISH assays were performed using a Fluorescent In Situ Hybridization Kit (RiboBio, China) according to the protocol. Cy3-labeled SNHG6 probes were designed and synthesized by RiboBio (China).

### Subcellular fractionation

The nuclear and cytosolic fractions were separated using a PARIS Kit (Invitrogen, USA) according to the manufacturer’s instructions.

### RNA pull-down assay

SNHG6 template DNA was transcribed in vitro with Biotin RNA Labeling Mix and T7 RNA polymerase (Roche, Switzerland) and purified with an RNeasy Mini Kit (Qiagen, USA) according to the manufacturer’s instructions. RNA-bound beads were incubated with total cell lysates of HCT-116, and eluted RNA was purified and assessed by qRT-PCR.

### RNA immunoprecipitation assay (RIP)

An EZ Magna RNA immunoprecipitation Kit (Millipore, USA) was used according to the manufacturer’s guidelines. Briefly, HCT-116 cells were lysed in RIP lysis buffer. Magnetic beads were preincubated with anti-AGO2 or IgG antibody for 30 min at room temperature, and the cell lysates were immunoprecipitated with beads for 6 h at 4 °C. Then, the immunoprecipitated RNA was purified and detected by qRT-PCR. The antibody information is listed in Additional file 1: Table S3.

### Gene set enrichment analysis

Gene set enrichment analysis (GSEA) was used to explore the pathways and gene sets associated with SNHG6 in colorectal cancer. Gene expression profiles of 481 colorectal cancer samples were downloaded from TCGA dataset. According to the SNHG6 expression level, the top 25 % and the bottom 25 % of samples were grouped as the high SNHG6 group and low SNHG6 group respectively. GSEA v3.0 was used to determine whether the members of the gene sets from the MSigDB database were randomly distributed at the top or bottom of the ranking [21]. The number of permutation was 1000, and the threshold for the nominal *P* value was set to 0.05. If most members of a gene set were positively or negatively related to SNHG6, the set was considered to be associated with SNHG6.

### Statistical analysis

All statistical analyses were performed using SPSS 18.0 (SPSS, USA) and GraphPad Prism 6 (GraphPad, USA) software. Chi-square test was used to analyze the different distribution of clinical variables. Differences in the level of gene expression were analyzed using Student’s *t* test. Univariate and multivariate Cox proportional hazards regression models were used to analyze potential factors associated with prognosis. Overall survival was estimated with the Kaplan–Meier method, and the log-rank test was employed to evaluate differences. For in vitro and in vivo experiments, a *t* test or analysis of variance was used to evaluate the differences between different groups. All *P* values were two-tailed, and *P* < 0.05 was considered statistically significant. All data are presented as the mean ± standard deviation (SD) from at least three independent replicates.

A complete description of the methods, including cell growth and colony formation assays, 5-Ethynyl-20-deoxyuridine (EdU), flow cytometry, protein extraction and western blot, Immunohistochemistry (IHC) and immunofluorescence (IF), wound healing assay, transwell migration and matrigel invasion assays and TUNEL assay are available in Additional file 2: Supplemental materials and methods.

## Results

### SNHG6 expression is upregulated in colorectal cancer samples and high SNHG6 expression predicates poor prognosis

**Fig. 1:**
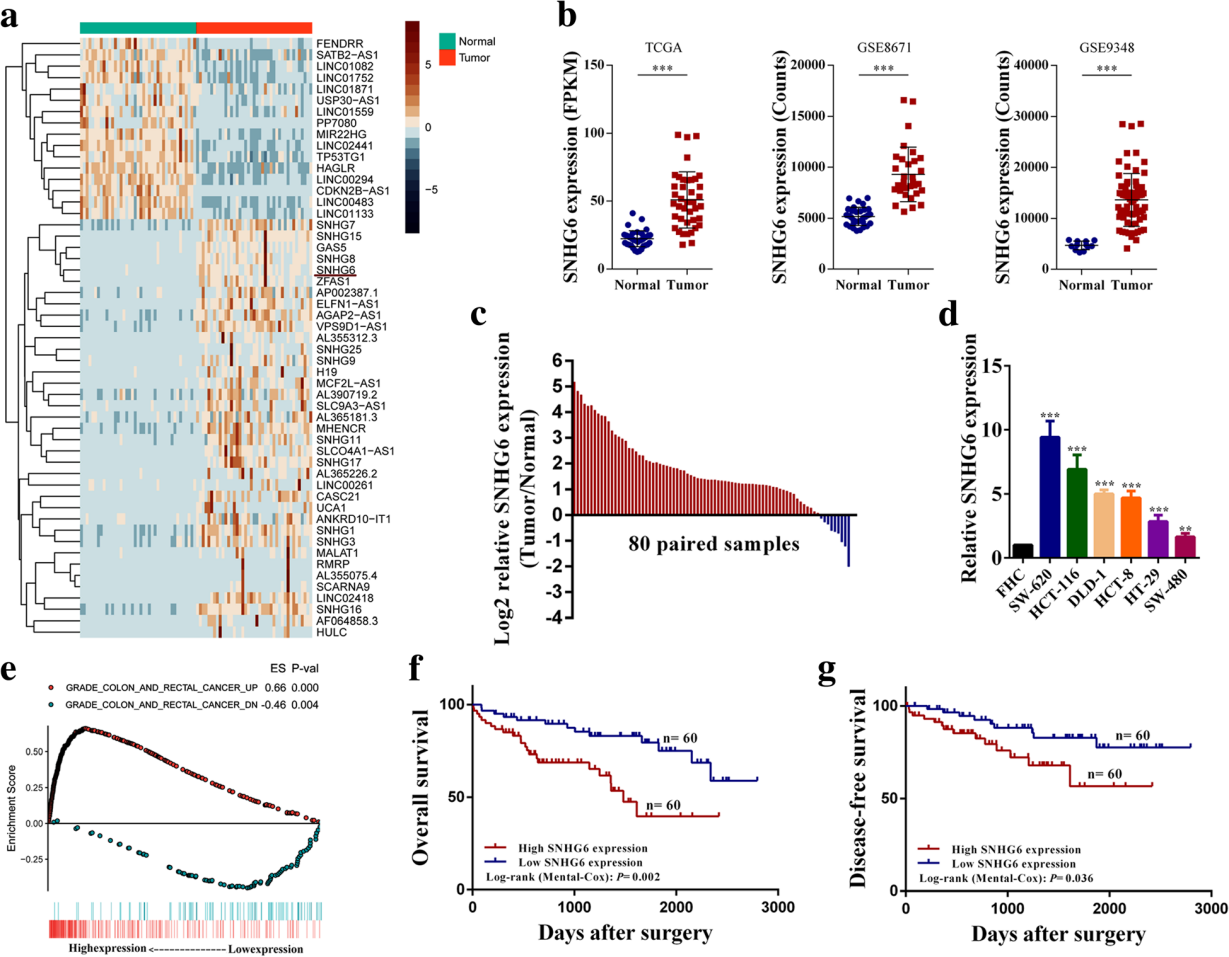
SNHG6 expression is upregulated in colorectal cancer and high SNHG6 expression predicts poor prognosis. **a** Hierarchical cluster heat map of aberrantly expressed lncRNAs in CRC generated from RNA sequencing data from the TCGA database. Red in the heat map denotes upregulation, while blue denotes downregulation. The red line indicates SNHG6. **b** Expression of SNHG6 in TCGA, GSE8671, and GSE9348 CRC cohorts. **c** qRT-PCR analysis of SNHG6 expression in 80 pairs of colorectal cancer and corresponding normal tissues. **d** SNHG6 expression in CRC cell lines (DLD-1, HCT-116, HT-29, SW-620, HCT-8, and SW-480) compared with normal colorectal epithelial cell line FHC detected by qRT-PCR. **e** Gene set enrichment analysis results of were plotted to visualize the correlation between the expression of SNHG6 and genes dysregulated in colorectal cancer (GRADE_COLON_AND_RECTAL_CANCER_DN and GRADE_COLON_AND_RECTAL_CANCER_UP). **f** Kaplan–Meier survival analysis of CRC patients’ overall survival based on their SNHG6 expression, a tissue sample whose threshold cycle (CT) value of SNHG6 minus CT value of GAPDH less than − 5.56 was defined as high SNHG6 expression (*n* = 120, *P* = 0.002). **g** Kaplan–Meier survival analysis of CRC patients’ disease-free survival based on their SNHG6 expression, a tissue sample whose threshold cycle (CT) value of SNHG6 minus CT value of GAPDH less than − 5.56 was defined as high SNHG6 expression (*n* = 120, *P* = 0.036). **P < 0.01 and ***P < 0.001

We first analyzed the lncRNA expression profiles of CRC tissue and the adjacent normal tissue samples in The Cancer Genome Atlas (TCGA) [22]. Many abnormally expressed lncRNAs were identified. SNHG6 ranked as one of the top remarkably upregulated lncRNAs with relatively high abundance (Fig. 1a). We then analyzed microarray datasets of two independent CRC cohorts and found that SNHG6 expression was also upregulated (Fig. 1b). Besides, we detected SNHG6 expression in 80 paired colorectal cancer tissue and adjacent normal tissue samples by qRT-PCR, and SNHG6 expression was significantly upregulated in 88.8% (71 of 80 paired) of the colorectal cancer tissue samples (Fig. 1c). In addition, we analyzed SNHG6 expression in human colorectal cancer cell lines. As shown in Fig. 1d, SNHG6 expression was upregulated in all six colorectal cancer cell lines (SW-620, HCT-116, DLD-1, HCT-8, HCT-29, and SW-480) compared with the human colorectal epithelial cell line FHC (Fig. 1d). To explore the clinical relevance of SNHG6 in CRC, we divided the enrolled patients into two groups according to SNHG6 expression. As shown in Table 1, statistical analysis demonstrated that SNHG6 expression was correlated with tumor invasion depth (*P* = 0.010), distant metastasis (*P* = 0.001), lymph node metastasis (*P* = 0.013), and TNM stage (*P* = 0.008). We also analyzed SNHG6 expression in different CRC stages using TCGA data, and advanced stage tumors had higher SNHG6 expression (Additional file 3: Figure S1a). GSEA also revealed significant relations between the expression of dysregulated genes in CRC and SNHG6 (Fig. 1e). In addition, Kaplan–Meier survival analysis showed that high SNHG6 expression was significantly correlated with poor overall survival (*P* = 0.002) and disease-free survival (*P* = 0.036, Fig. 1h, i). The same result was observed in two independent CRC cohorts from the R2 database (http://hgserver1.amc.nl/cgi-bin/r2/main.cgi) (Additional file 3: Figure S1b). To further evaluate the prognostic value of SNHG6, both univariate and multivariate analyses were performed. The univariate analysis results indicated that SNHG6 expression (HR = 3.24, 95% CI = 1.84–5.86, *P* < 0.001), TNM stage (HR = 2.72, 95% CI = 1.48–4.88, *P* < 0.001), and distant metastasis status (HR = 4.82, 95% CI = 2.52–8.96, *P* < 0.001) were prognostic factors. In addition, the multivariate analysis of the three prognosis factors revealed that SNHG6 expression was an independent prognostic biomarker (HR = 2.48, 95% CI = 1.60–5.86, *P* = 0.002) for colorectal cancer (Table 2). These results reveal that SNHG6 upregulation may play a critical role in the development and progression of colorectal cancer.

**Table 2:** Univariate and multivariate analysis of clinic pathologic factors for overall survival in 120 patients with CRC

Risk factors	Univariate analysis		Multivariate analysis	
	HR (95% CI)	*P* value^a^	HR (95% CI)	*P* value^a^
SNHG6 expression (low vs. high expression)	3.24 (1.84–5.86)	< *0.001*	2.48 (1.60–5.86)	*0.002*
TNM stage (I/II vs. III/IV)	2.72 (1.48–4.88)	< *0.001*	1.24 (0.48–2.74)	0.658
Tumor invasion depth (T1*/*T2 vs. T3*/*T4)	1.62 (0.82–2.84)	0.184		
Lymph node metastasis (N0 vs. N1 or above)	1.62 (0.77–2.81)	0.622		
Distant metastasis (M0 vs. M1)	4.82 (2.52–8.96)	< *0.001*	4.06 (1.83–8.87)	*0.001*
Age (≤ 60 vs. > 60)	0.97 (0.58–1.66)	0.892		
Gender (male vs. female)	0.92 (0.52–1.60)	0.584		

*HR* hazard ratio, *CI* confidential interval, vs. versus

^a^Statistical significant results (in italics)

### High SNHG6 expression is partly due to DNA copy number gains and SP1 activation in CRC

**Fig. 2:**
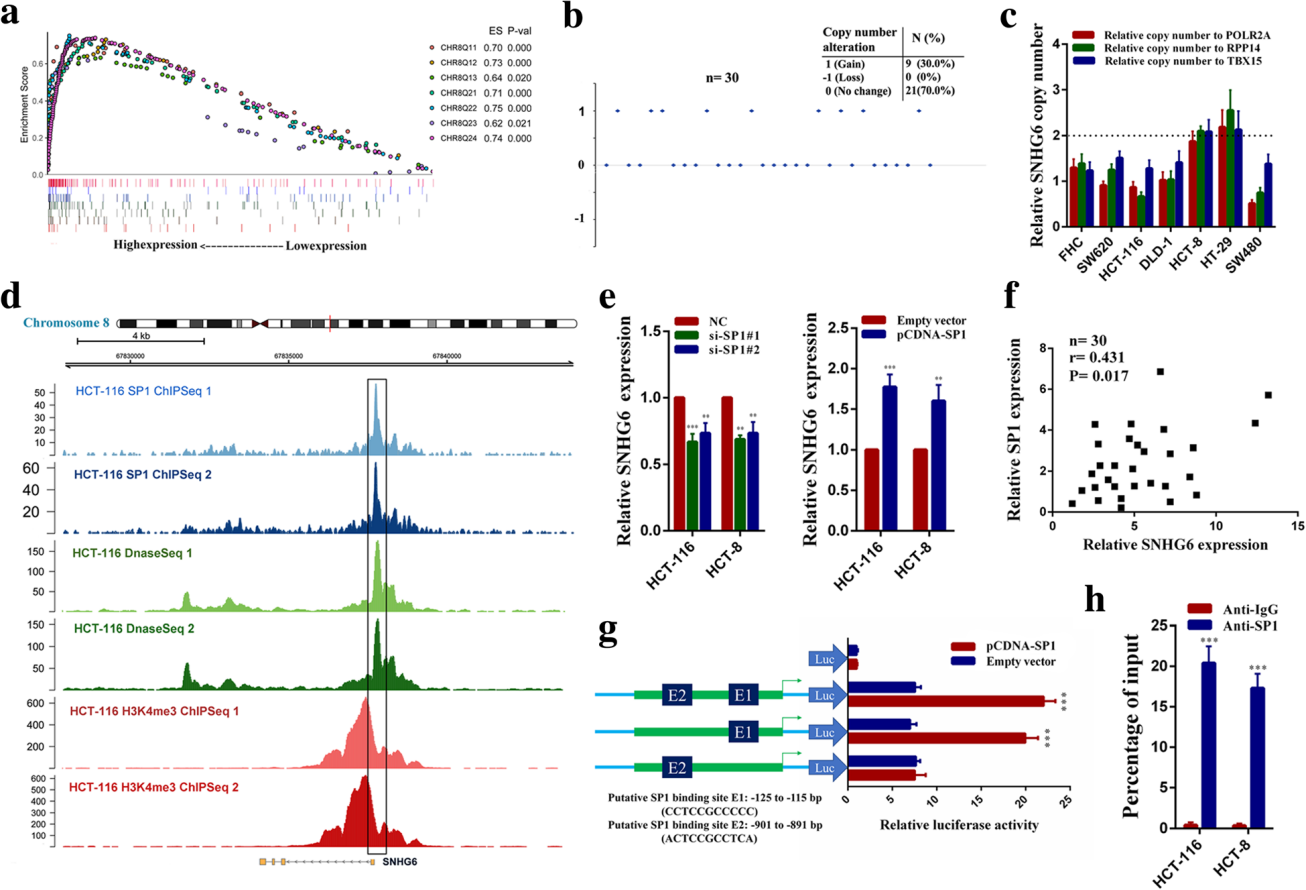
DNA copy number gains and SP1 activation induce high SNHG6 expression in CRC. **a** GSEA results were plotted to visualize the correlation between the expression of SNHG6 and genes in adjacent chromosomal regions (CHR8Q11, CHR8Q12, CHR8Q13, CHR8Q21, CHR8Q22, CHR8Q23, and CHR8Q24). **b** SNHG6 genomic copy numbers in 30 CRC tissue samples. **c** SNHG6 genomic copy numbers in CRC cell lines and the normal colorectal epithelial cell line FHC. **d** Analysis of SP1 ChIP-seq, H3K4me3 ChIP-seq, and DnaseI-seq data of HCT-116 cells in the SNHG6 locus. **e** SNHG6 expression was detected by qRT-PCR in HCT-116 and HCT-8 cells transfected with the SP1 siRNAs or SP1 overexpression vector. **f** The correlation between the SP1 and SNHG6 expression levels were analyzed in 30 paired colorectal cancer samples (*n* = 30, *r* = 0.431, *P* = 0.017). **g** Luciferase reporter assays were used to determine the SP1 binding sites on the SNHG6 promoter region. **h** ChIP assays were performed to detect SP1 occupancy in the SNHG6 promoter region. **P < 0.01 and ***P < 0.001

We next explored which factors induced high SNHG6 expression in CRC. SNHG6 is located on chromosome 8q13, a region with frequent copy number amplification in CRC. We speculated DNA copy number gains might be responsible for its upregulation. GSEA results also indicated that SNHG6 expression was positively correlated with the expression of genes in adjacent chromosomal regions (Fig. 2a). We then detected the genomic copy number levels of SNHG6 in 30 CRC tissue samples, and copy number gains was identified in 30% (9 of 30) colorectal cancer tissues (Fig. 2b). We also detected the SNHG6 genomic copy number levels in CRC cell lines, and SNHG6 copy number gains were observed in HCT-8 and HT-29 cells (Fig. 2c). Besides, we explored transcription factors that could potentially upregulate SNHG6 in CRC. We used the JASPAR CORE database to search for transcription factor binding sites in the SNHG6 promoter [23]. We found two putative SP1 binding sites: E1 (CCTCCGCCCCC, − 125 bp to − 115 bp) and E2 (ACTCCGCCTCA, − 901 bp to − 891 bp), got relatively high scores. We then analyzed SP1 ChIP-Seq data of HCT-116 downloaded from the Encyclopedia of DNA Elements (ENCODE) database. As shown in Fig. 2d, SP1 was highly enriched in the SNHG6 promoter region. We then silenced SP1 in HCT-116 and HCT-8 cells, and SNHG6 expression was decreased. In contrast, SP1 overexpression increased SNHG6 expression (Additional file 3: Figure S2a and Fig. 2e). In addition, we found SNHG6 expression was elevated in samples with high SP1 expression and that SNHG6 expression was positively correlated with SP1 expression in CRC tissues (Fig. 2f and Additional file 3: Figure S2b). Besides, luciferase reporter assays showed SP1 mainly bound to the E1 site of SNHG6 promoter (Fig. 2g). Furthermore, ChIP assays performed on E1 region of the SNHG6 promoter indicated SP1 interacted with the SNHG6 promoter region directly (Fig. 2h). Overall, the above results indicate that the upregulation of SNHG6 in CRC is partly due to SNHG6 genomic copy number gains and SP1 activation.

### SNHG6 promotes CRC cell growth in vitro

**Fig. 3:**
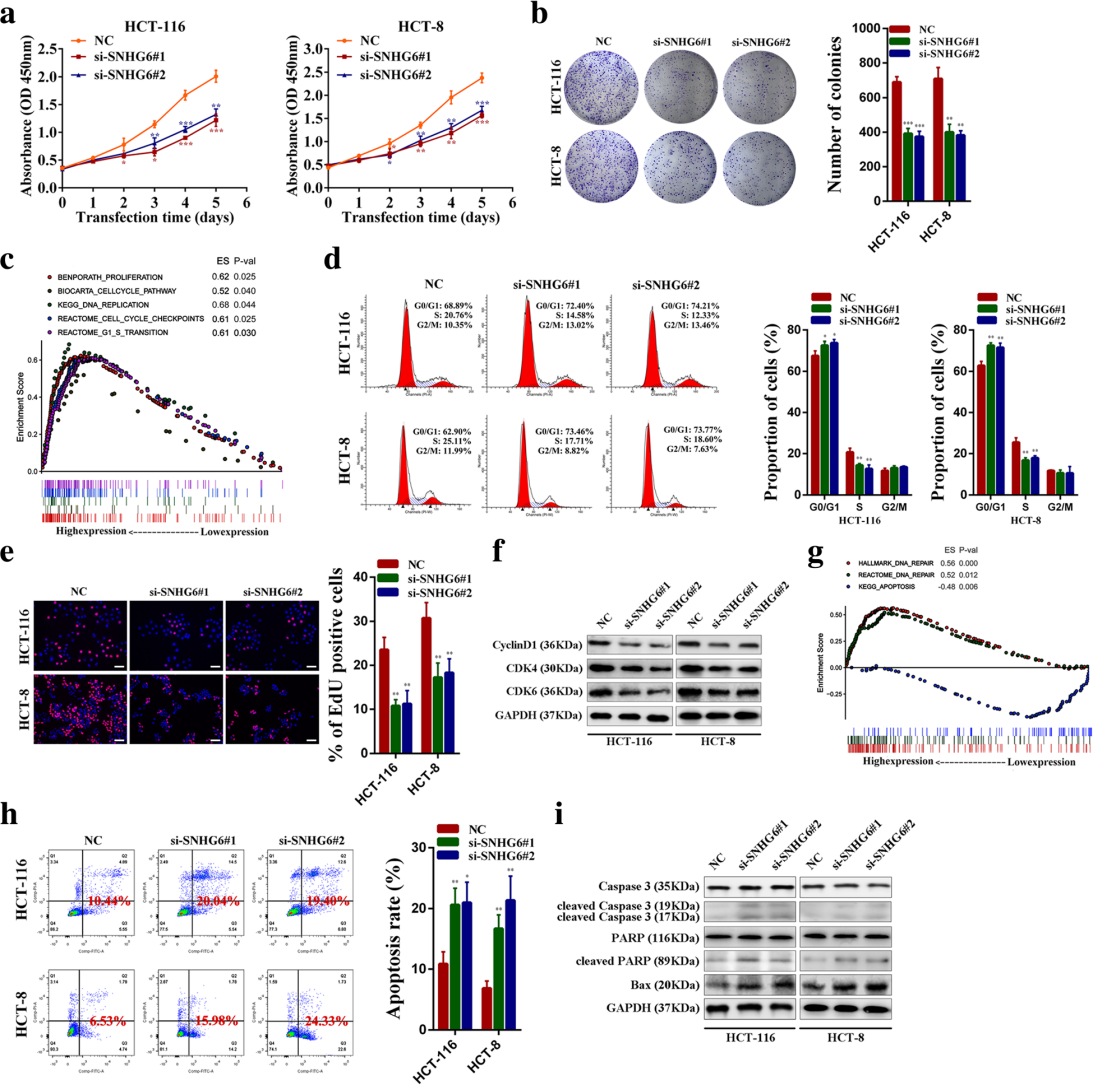
SNHG6 affects CRC cell growth in vitro. **a** HCT-116 and HCT-8 cells transfected with the SNHG6 siRNAs were subjected to the CCK-8 assay. **b** HCT-116 and HCT-8 cells transfected with SNHG6 siRNAs were seeded into 6-well plates. The number of colonies was counted on the 14th day after seeding. **c** GSEA results were plotted to visualize the correlation between the expression of SNHG6 and genes related to cell proliferation (BENPORATH_PROLIFERAYION, BIOCARTA _CELLCYCLE_PATHWAY, KEGG_DNA_REPLICATION, REACTOME_CELL _CYCLE_ CHECKPOINTS, and REACTOMT _G1_S_TRANSITION). **d** Flow cytometric cell cycle distribution assays to detect the proportion of CRC cells in the G1, S, and G2/M phases after the transfection of SNHG6 siRNAs. **e** EdU assays were used to determine the cell proliferation ability of SNHG6 siRNAs transfected cells. **f** Cell cycle-related proteins CyclinD1, CDK4, and CDK6 were detected by western blotting following SNHG6 silencing. **g** GSEA results were plotted to visualize the correlation between the expression of SNHG6 and genes related to cell apoptosis (HALLMARK_DNA_REPAIR, REACTOME_DNA_REPAIR, and KEGG _APOPTOSIS). **h** The effect of SNHG6 knockdown on cell apoptosis was analyzed by flow cytometric cell apoptosis assays. **i** The apoptosis-related proteins caspase-3, cleaved caspase-3, PARP, cleaved PARP and Bax were detected by western blotting after SNHG6 knockdown. Scale bar = 50 μm. *P < 0.05, **P < 0.01 and ***P < 0.001

To further elucidate the role of SNHG6 in CRC, we first designed two independent small interfering RNAs (siRNAs) targeting SNHG6. As shown in Additional file 3: Figure S3a and b, these siRNAs could silence its expression and SNHG6 overexpression vectors could increase its expression efficiently. Then, CCK-8 assays demonstrated that SNHG6 knockdown inhibited HCT-116 and HCT-8 cell growth and SNHG6 overexpression promoted HCT-116 and HCT-8 cell growth significantly (Fig. 3a and Additional file 3: Figure S3c). Similarly, clone formation assays showed that SNHG6 knockdown decreased the clone-forming ability of CRC cells and SNHG6 overexpression increased the clone-forming ability of CRC cells (Fig. 3b and Additional file 3: Figure S3d). GSEA results revealed significant relations between the expression of cell cycle-related genes and SNHG6 in CRC (Fig. 3c). We then employed flow cytometry cell cycle assays and Ethynyl deoxy Uridine (EdU) dye assays to detect cell cycle progression and proliferation rates of SNHG6-silenced CRC cells. As we speculated, SNHG6 knockdown could induce cell cycle arrest and decrease the cell proliferation rate (Fig. 3d, e). In addition, western blotting results showed that the expression levels of cell cycle-related proteins cyclin D1, CDK4, and CDK6 were all decreased in si-SNHG6 transfected CRC cells (Fig. 3f). Moreover, GSEA results also indicated that SNHG6 expression exhibited significant relations with the expression of DNA repair and apoptosis-related genes in CRC (Fig. 3g). Flow cytometry cell apoptosis analysis showed SNHG6 knockdown significantly increased the proportion of apoptotic cells (Fig. 3h). Besides, SNHG6 silencing increased the expression of apoptosis-related proteins cleaved caspase-3, cleaved PARP, and Bax (Fig. 3i). In addition, we found that caspase-3 inhibitor Z-DEVD-FMK treatment (50 μM, 24 h) could partially abolish apoptosis of SNHG6 knockdown CRC cells (Additional file 3: Figure S3e). Taken together, these results demonstrate that SNHG6 can affect CRC cell growth by facilitating cell cycle progression and inhibiting cell apoptosis.

### SNHG6 promotes CRC cell migration and invasion in vitro

**Fig. 4:**
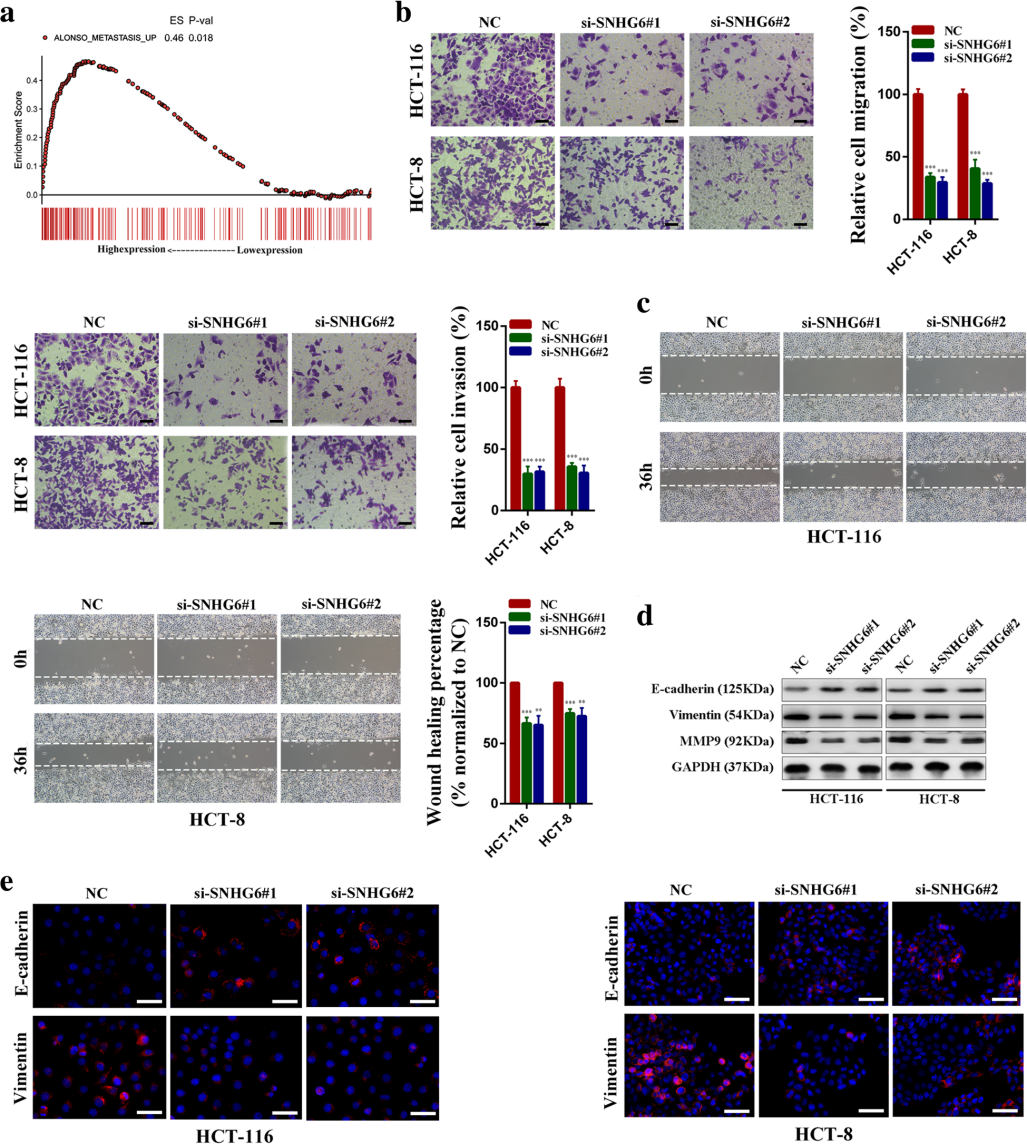
SNHG6 affects CRC cell migration and invasion in vitro. **a** GSEA results were plotted to visualize the correlation between the expression of SNHG6 and genes related to cancer metastasis (ALONSO_METASTASIS). **b** Transwell assays were used to determine the invasion and migration abilities of SNHG6 siRNAs transfected cells. **c** Representative images of wound healing assays performed using HCT-6 cells and HCT-8 cells after SNHG6 silenced. **d** The metastasis-related proteins E-cadherin, vimentin, and MMP9 were detected by western blotting after SNHG6 knockdown. **e** The E-cadherin and vimentin protein levels were detected by immunofluorescence after SNHG6 knockdown. Scale bar = 50 μm. **P < 0.01 and ***P < 0.001

Subsequently, we explored the effects of SNHG6 on CRC metastasis. GSEA results indicated that SNHG6 expression was significantly correlated with the expression of metastasis-related genes (Fig. 4a). Transwell assays demonstrated that SNHG6 knockdown inhibited the migration and invasion abilities of HCT-116 and HCT-8 cells, and SNHG6 overexpression increased the migration and invasion abilities of HCT-116 and HCT-8 cells (Fig. 4b and Additional file 3: Figure S3f). The wound-healing assays showed that CRC cells transfected with SNHG6 siRNAs underwent slower scratch wound closure than the negative control (NC) cells (Fig. 4c). Besides, western blotting results showed that the protein level of the epithelial marker E-cadherin was markedly increased in SNHG6-silenced CRC cells. Conversely, the expression of the mesenchymal markers vimentin and MMP-9 was decreased (Fig. 4d). The same results were observed in immunofluorescence assays (Fig. 4e).

### SNHG6 promotes CRC cell growth and metastasis in vivo

**Fig. 5:**
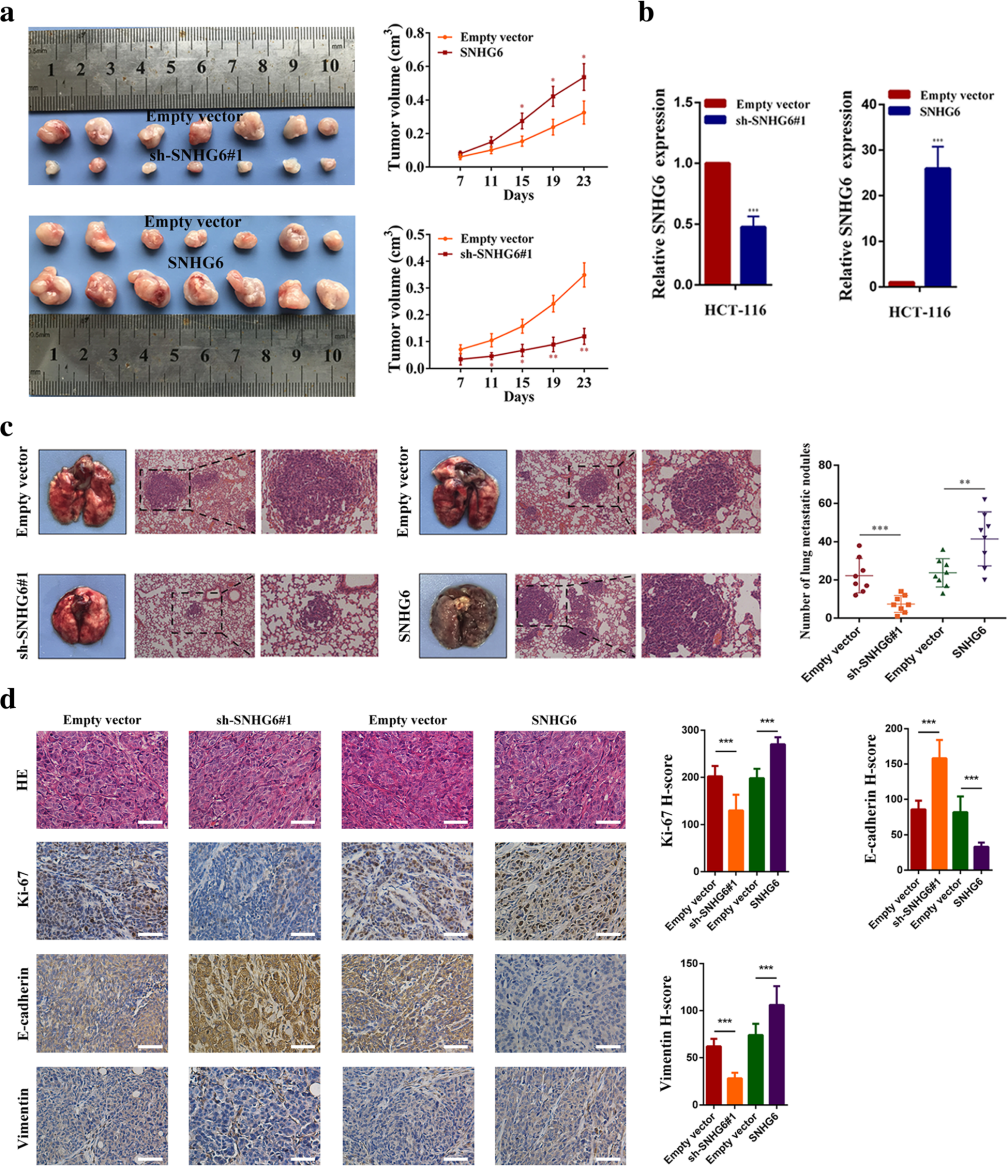
SNHG6 promotes CRC growth and metastasis in vivo. **a** Representative image of tumors formed in nude mice from empty vector, sh-SNHG6#1 vector and SNHG6 overexpression vector groups, and the tumor volume growth curves of different groups. **b** SNHG6 expression was detected in tumors from the different groups of mice by qRT-PCR. **c** Left panel, representative images of the gross lesion in lung tissues and hematoxylin and eosin (HE) staining of metastatic nodules in the lungs from the different groups. Right panel, the statistical result of metastatic nodule numbers in the lungs from the different groups. **d** Left panel, representative images for HE-staining, Ki67, E-cadherin, and vimentin immunostaining of tumor samples from the different groups. Right panel, the statistical results of these immunochemistry assays. Scale bar = 50 μm. *P < 0.05, **P < 0.01, and ***P < 0.001

To evaluate the biological functions of SNHG6 in vivo, HCT-116 cells stably transfected with sh-SNHG6#1, SNHG6, or the corresponding empty vectors were subcutaneously or intravenously injected into nude mice. We found that tumor lumps in the sh-SNHG6#1 group were significantly smaller than those in the empty vector group. Conversely, the tumor volumes in the SNHG6 overexpressing group were larger than those in the empty vector group (Fig. 5a). At the end of this experiment, the mice were sacrificed, and we measured SNHG6 expression in each group. As expected, tumors formed from SNHG6 knockdown cells had lower SNHG6 expression and formed from SNHG6 overexpressing cells had higher SNHG6 expression (Fig. 5b). For lung metastasis, the number of metastatic nodules in the sh-SNHG6#1 group was lower than that in the vector control group. Accordingly, the number of metastatic nodules was increased in the SNHG6 overexpressing group (Fig. 5c). Besides, tumor tissues collected from the sh-SNHG6#1 group had lower Ki67-positive rates, whereas tissues collected from the SNHG6 overexpressing group had higher Ki67-positive rates than those from the corresponding control group. We also detected E-cadherin and vimentin expression in xenograft tumor tissues by immunohistochemistry. We found the E-cadherin expression was upregulated in the sh-SNHG6#1 group and downregulated in the SNHG6 overexpressing group. Vimentin expression exhibited the opposite trend (Fig. 5d). In addition, the terminal transferase dUTP nick end labeling (TUNEL) assays demonstrated that the tissues form the sh-SNHG6#1 group had higher cell apoptotic rates, whereas the tissues from the SNHG6 overexpressing group had lower cell apoptotic rates (Additional file 3: Figure S4a). Taken together, these results indicate that SNHG6 promotes tumor growth and metastasis in vivo, which is consistent with its functions in vitro.

### SNHG6 acts as a molecular sponge for miR-26a/b and miR-214 in CRC

**Fig. 6:**
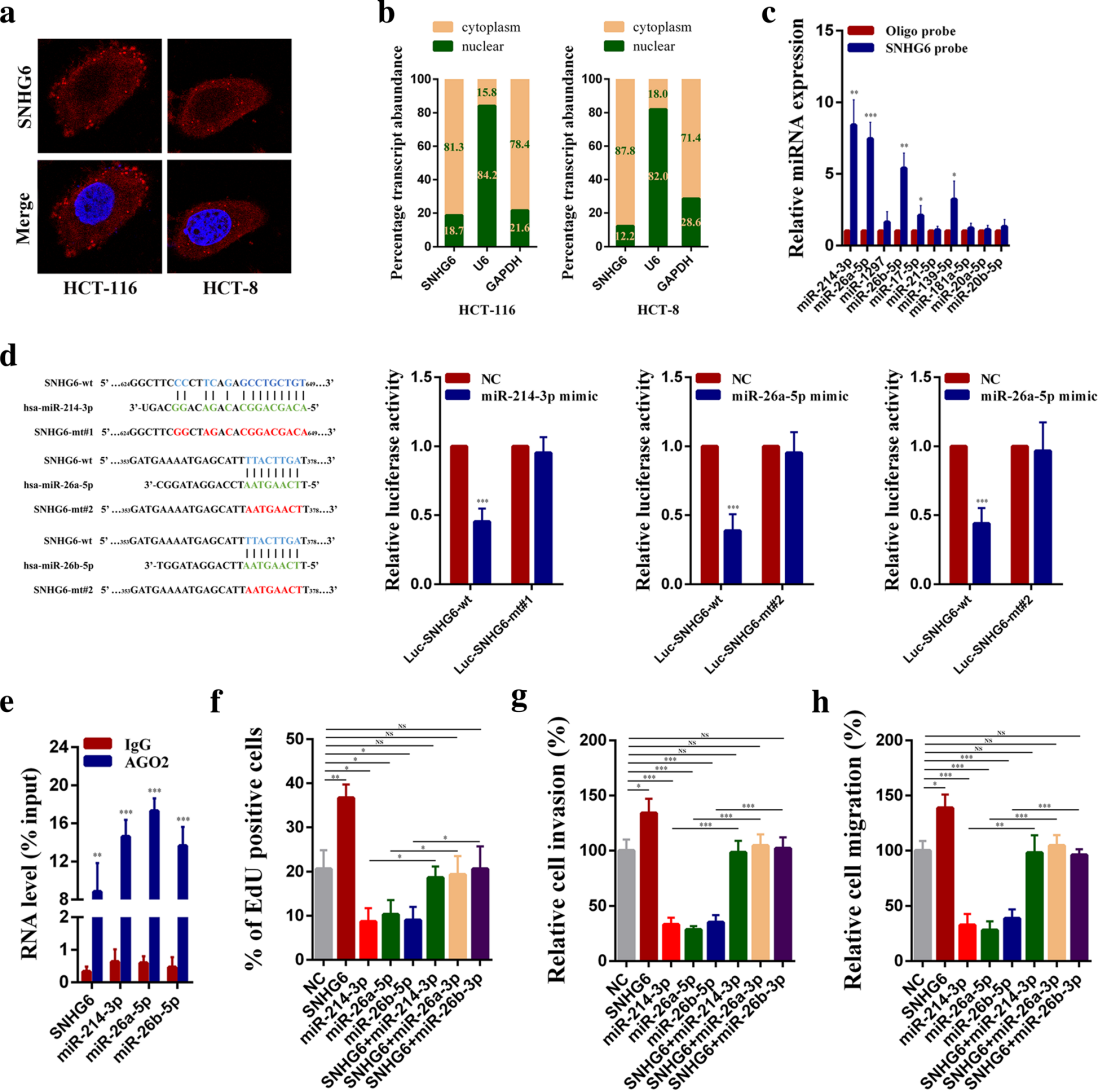
SNHG6 acts as a sponge for miR-214, miR-26a, and miR-26b in the cytoplasm. **a** Representative FISH images showed the expression of SNHG6 in HCT-116 and HCT-8 cells (red). Nuclei were stained by DAPI (blue). **b** Relative SNHG6 expression levels in nuclear and cytosolic fractions of HCT-116 and HCT-8 cells. Nuclear controls: U6, cytosolic controls: GAPDH. **c** The relative expression of candidate microRNAs which could potentially bind to SNHG6 were quantified by qRT-PCR after the biotinylated-SNHG6 pull-down assays in HCT-116 cells. **d** Dual luciferase reporter assays were conducted with wild-type and mutant-type (putative binding sites for miR-214, miR-26a, or miR-26b were mutated) luciferase report vectors. Left panel, sequence alignment of miR-214, miR-26a, or miR-26b and their predicted binding sites (green) of SNHG6. Predicted microRNA target sequence (blue) in SNHG6 (Luc-SNHG6-wt) and positions of mutated nucleotides (red) in SNHG6 (Luc-SNHG6-mt#1 and Luc-SNHG6-mt#2). **e** RNA immunoprecipitation with an anti-Ago2 antibody was used to assess endogenous Ago2 binding to RNA, IgG was used as the control. The levels of SNHG6, miR-214, miR-26a, and miR-26b were determined by qRT–PCR and presented as fold enrichment in Ago2 relative to input. **f** EdU assays showed that overexpression of SNHG6 promotes CRC cells proliferation. Overexpression of miR-214, miR-26a, or miR-26b inhibits cancer cell proliferation. Co-transfecting miR-214, miR-26a, or miR-26b mimics with SNHG6 plasmids abolished the increased proliferation rates. **g**, **h** Transwell assays showed that overexpression of SNHG6 promotes CRC cells migration and invasion. Overexpression of miR-214, miR-26a, or miR-26b inhibits CRC cells migration and invasion. Co-transfecting miR-214, miR-26a, or miR-26b mimics with SNHG6 plasmids abolished the increased migration and invasion abilities. Scale bar = 50 μm. *P < 0.05, **P < 0.01, and ***P < 0.001

To elucidate the potential molecular mechanisms through which SNHG6 contributes to the progression of CRC, we first examined its localization in CRC cells, because the functions of a lncRNA depended on its subcellular distribution [24]. Through FISH and subcellular fractionation assays, we identified that SNHG6 was mostly expressed in the cytoplasm (Fig. 6a, b). Many cytoplasmic lncRNAs have been reported to act as ceRNAs by competitively binding microRNAs. Thus, to demonstrate whether SNHG6 acts as a ceRNA, we first used miRcode software to explore miRNAs that could potentially target SNHG6 [25]. We found that a set of microRNAs (miR-214-3p, miR-26a-5p, miR-26b-5p, miR-1297, miR-17-5p, miR-21-5p, miR-139-5p, miR-181a-5p, miR-20a-5p, and miR-20b-5p) were predicted to have a high probability of combining to SNHG6. We next conducted RNA pull-down experiments with biotin-labeled SNHG6 in HCT-116 cells. As shown in Fig. 6c, miR-214-3p, miR-26a-5p, miR-26b-5p, miR-17-5p, and miR-139-5p could be pulled down by SNHG6. To further identify which microRNAs could interact with SNHG6 directly, we constructed corresponding dual luciferase reporter vectors for these five microRNAs. Dual luciferase reporter assays in HCT-116 cells indicated that only overexpression of miR-214-3p, miR-26a-5p, and miR-26b-5p could significantly decrease the luciferase activity (Additional file 3: Figure S5b). The efficiencies of mimics and inhibitors of the microRNAs are shown in Additional file 3: Figure S5a. In addition, to examine whether miR-214-3p, miR-26a-5p, and miR-26b-5p could bind to the predicted target sites in SNHG6, we constructed wild-type and mutant-type (putative binding sites for the microRNAs were mutated) luciferase reporter vectors of SNHG6. As expected, co-transfection of the wild-type SNHG6 luciferase vector (Luc-SNHG6-wt) with the miR-214-3p, miR-26a-5p, or miR-26b-5p mimics, but not the mutant SNHG6 vector (Luc-SNHG6-mt#1, Luc-SNHG6-mt#2), significantly decreased the luciferase activity (Fig. 6d). We also found overexpressing SNHG6 could reduce miR-214-3p, miR-26a-5p, and miR-26b-5p expression and knockdown of SNHG6 could increase these microRNA expressions significantly (Additional file 3: Figure S5c). Besides, correlation analysis revealed that there were negative correlations between the expression levels of SNHG6 and these microRNAs in 30 CRC tissues (Additional file 3: Figure S5d). Moreover, RIP assay results showed SNHG6, miR-214-3p, miR-26a-5p, and miR-26b-5p were all significantly enriched in AGO2-containing micro-ribonucleoprotein complexes, suggesting that the AGO2 protein bound directly to SNHG6, miR-214-3p, miR-26a-5p, and miR-26b-5p in CRC cells (Fig. 6e). The above results demonstrate that SNHG6 acts as a molecular sponge for miR-26a/b and miR-214 in CRC cells.

We next explored whether oncogenic functions of SNHG6 were dependent on sponging miR-214-3p, miR-26a-5p, and miR-26b-5p. The results of CCK-8 assays showed that miR-214-3p, miR-26a-5p, or miR-26b-5p downregulation could rescue the growth inhibition of HCT-116 cells caused by SNHG6 knockdown (Additional file 3: Figure S6a). In addition, by performing EdU assays, we found that SNHG6 overexpression could significantly increase HCT-116 cell proliferation rates compared with cells containing empty vectors and this increase could be eliminated when miR-214-3p, miR-26a-5p, or miR-26b-5p were transfected (Fig. 6f and Additional file 3: Figure S6b). Similarly, transwell assays showed that SNHG6 overexpression could significantly increase the migration and invasion abilities of HCT-116 cells and these increases could again be partially abolished when miR-214-3p, miR-26a-5p, or miR-26b-5p were transfected (Fig. 6g, h, Additional file 3: Figure S6c and d).

Taken together, these results indicate that SNHG6 promotes CRC progression by serving as a ceRNA for miR-214-3p, miR-26a-5p, and miR-26b-5p.

### SNHG6 functions as a ceRNA to regulate EZH2 expression in CRC

**Fig. 7:**
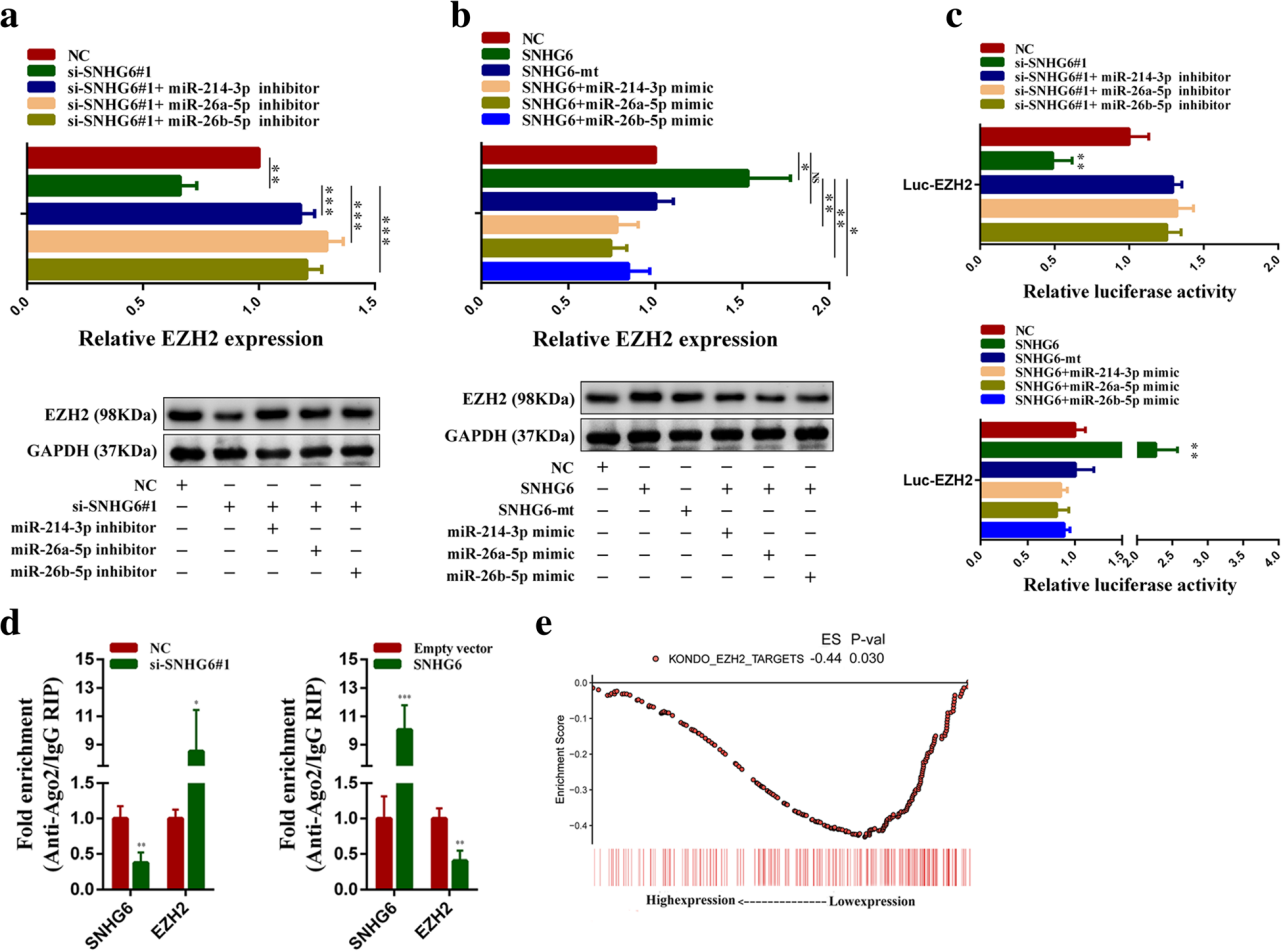
SNHG6 regulates expression of the common target of miR-214, miR-26a, or miR-26b, EZH2. **a**, **b** EZH2 expression was detected by qRT-PCR or western blotting in HCT-116 cells with indicated treatment. SNHG6-mt contains mutations at the putative miR-214-3p, miR-26a-5p, and miR-26b-5p binding sites. **c** Luciferase activity of Luc-EZH2 reporters which contained the 3′-untranslated region (3′-UTR) of EZH2 with indicated treatment in HCT-116 cells. **d** RIP assay of the enrichment of Ago2 on SNHG6 and EZH2 transcripts relative to IgG in HCT-116 cells transfected with SNHG6 overexpression vectors or siRNAs. **e** Results of GSEA were plotted to visualize the correlation between the expression of SNHG6 and genes related with EZH2 targets (KONDO_EZH2 _TARGETS). Scale bar = 50 μm. *P < 0.05, **P < 0.01, and ***P < 0.001

The functions of microRNAs rely on their downstream targets. Through literature review, we found that the oncogene EZH2 was reported to be a common target of these microRNAs. Our results also demonstrated that EZH2 expression was elevated in CRC tissues and that the overexpression of miR-214-3p, miR-26a-5p, or miR-26b-5p could decrease EZH2 expression in HCT-116 cells (Additional file 3: Figure S7a and b). Thus, we speculated that EZH2 might be involved in tumor-promoting functions of SNHG6. By expression analysis in HCT-116 cells, we found that when SNHG6 was silenced, EZH2 was downregulated. Besides, the inhibition of miR-214-3p, miR-26a-5p, or miR-26b-5p in SNHG6-silenced cells reversed the decrease of EZH2. Western blotting assays also showed that the inhibition of miR-214-3p, miR-26a-5p, or miR-26b-5p could rescue the EZH2 protein level decrease induced by SNHG6 knockdown (Fig. 7a). In addition, the overexpression of SNHG6 could upregulate EZH2, and transfection of the miR-214-3p, miR-26a-5p, or miR-26b-5p mimics abolished EZH2 increase in SNHG6-upregulated cells. To further verify that SNHG6 could regulate EZH2 expression by sponging these microRNAs, we constructed a SNHG6-mt vector, which contains mutations in the putative miR-214-3p, miR-26a-5p, and miR-26b-5p binding sites (Additional file 3: Figure S7c). As expected, transfecting the mutant SNHG6 plasmids into cells did not increase EZH2 expression. Meanwhile, the western blotting results were in agreement with the qPCR results (Fig. 7b). We then constructed luciferase reporter vectors Luc-EZH2 containing the 3′-untranslated region (3’-UTR) of EZH2. Dual luciferase reporter assays in HCT-116 cells showed that SNHG6 knockdown reduced Luc-EZH2 luciferase activity significantly, and the transfection of the miR-214-3p, miR-26a-5p, or miR-26b-5p inhibitors antagonized this decrease. We also found that the transfection of wild-type SNHG6 plasmids but not the SNHG6-mt plasmids could increase Luc-EZH2 luciferase activity, and the overexpression of miR-214-3p, miR-26a-5p, or miR-26b-5p could weaken the luciferase activity increase induced by ectopic SNHG6 expression (Fig. 7c). Besides, RIP assay results in SNHG6-silenced HCT-116 cells showed that the enrichment of Ago2 on SNHG6 was decreased and the enrichment of Ago2 on EZH2 was increased. Opposite changes were observed in SNHG6 overexpressed HCT-116 cells (Fig. 7d). Moreover, we observed a positive correlation between the expression levels of SNHG6 and EZH2 in 30 CRC tissues. The correlation between the expression levels of SNHG6 and EZH2 was confirmed in five independent CRC cohorts from the GEO database (Additional file 3: Figure S7d). The GSEA results also indicated a significant negative correlation between the expression of SNHG6 and the targets of EZH2 (Fig. 7e). In addition, immunohistochemistry assays showed that the EZH2 expression was significantly decreased in SNHG6 knockdown cells formed xenograft tumors and significantly increased in SNHG6 overexpression cells formed xenograft tumors (Additional file 3: Figure S7e). IHC assays also demonstrated that EZH2 protein levels were higher in high SNHG6 expression CRC tissues (Additional file 3: Figure S7f). Collectively, these results suggest that SNHG6 can release EZH2 by sequestering endogenous miR-214-3p, miR-26a-5p, and miR-26b-5p in CRC cells.

### EZH2 is responsible for the tumor-promoting effects of SNHG6

**Fig. 8:**
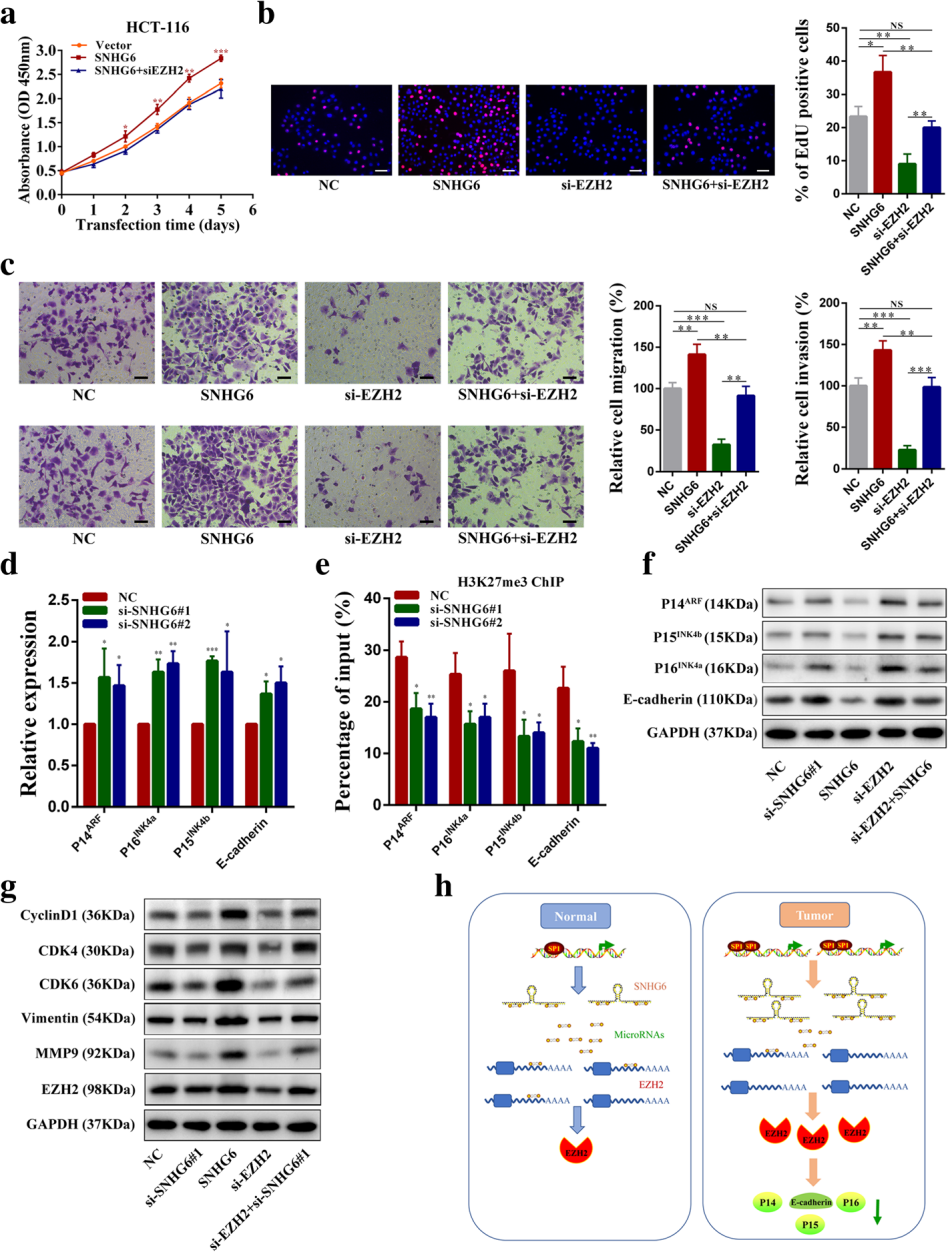
Tumor-promoting functions of SNHG6 is dependent on EZH2. **a** CCK-8 assays demonstrated that overexpression of SNHG6 promoted cancer cell growth. EZH2 knockdown could abolish growth promotion caused by SNHG6. **b** EdU assays showed that EZH2 knockdown abolished the increased proliferation rates of HCT-116 cells caused by SNHG6. **c** Transwell assays demonstrated that EZH2 knockdown abolished the increased abilities of migration and invasion caused by SNHG6. **d** Expression of P14^ARF^, P15^INK4b^, P16^INK4a^, and E-cadherin was detected by qRT-PCR in SNHG6-silenced HCT-116 cells. **e** ChIP assays revealed enrichment of H3K27me3 on promoter regions of P14^ARF^, P15^INK4b^, P16^INK4a^, and E-cadherin. **f** Expression of P14^ARF^, P15^INK4b^, P16^INK4a^, and E-cadherin was detected by western blotting in SNHG6-silenced HCT-116 cells. **g** The EZH2, cell cycle-related proteins, and metastasis-related proteins were detected by western blotting. **h** Schematic of the proposed mechanism of SNHG6 in CRC. Scale bar = 50 μm. *P < 0.05, **P < 0.01, and ***P < 0.001

To investigate whether SNHG6 exerted oncogene functions by modulating EZH2, we first examined the effect of EZH2 on SNHG6-induced promotion of cell growth. The CCK-8 assay results showed that silencing EZH2 could abolish the growth acceleration of CRC cells induced by SNHG6 overexpression, and the EdU assay results revealed that silence of EZH2 could impair the increase of cell proliferative rates induced by SNHG6 upregulation (Fig. 8a, b). In addition, the increased cell migration and invasion abilities in SNHG6 overexpressing CRC cells were reversed by EZH2 knockdown (Fig. 8c). Many studies have shown that EZH2 can transcriptionally repress the INK4B-ARF-INK4A tumor suppressor locus to drive cell cycle progression and promote epithelial-mesenchymal transition (EMT) by suppressing the epithelial marker E-cadherin. Therefore, we next explored whether SNHG6 influenced the expression of these EZH2 targets. As shown in Fig. 8d, SNHG6 knockdown increased P14^ARF^, P15^INK4b^, P16^INK4a^, and E-cadherin expression. Besides, ChIP assays revealed enrichment of the H3K27me3 modification induced by PRC2 was reduced in their promoter region after SNHG6 knockdown (Fig. 8e). Western blotting results showed that SNHG6 overexpression decreased the protein levels of P14^ARF^, P15^INK4b^, P16^INK4a^, and E-cadherin, and these decreases could be rescued by EZH2 silencing. In comparison, SNHG6 knockdown inhibited P14^ARF^, P15^INK4b^, P16^INK4a^, and E-cadherin expression in CRC cells (Fig. 8f). Moreover, SNHG6 overexpression increased the protein levels of cell cycle-related proteins and EMT related proteins, while SNHG6 silencing decreased them. Similarly, their increases induced by SNHG6 were impaired by EZH2 knockdown (Fig. 8g). Together, these data suggest that SNHG6 contributes to CRC progression through regulating EZH2 and its downstream targets.

## Discussion

In the human genome, in addition to microRNAs, which have strong regulatory and epigenetic modification functions, there are tens of thousands of other non-coding RNAs, including lncRNAs. lncRNAs were originally thought to be byproducts of RNA polymerase II transcription, genomic noises, and without biological function. However, accumulating evidence indicates that they can participate in numerous biological processes and play important roles in the genesis and development of diseases [26, 27]. Recent studies have reported that several lncRNAs are involved in tumorigenesis and progression of CRC. For example, Lan et al. found that the lncRNA OCC-1 played a tumor suppressive role in CRC by destabilizing HuR protein [28]. Lu et al. demonstrated that the lncRNA MIR100HG could mediate CRC cetuximab resistance by regulating miR-100 and miR-125b [29]. Ozawa et al. revealed that the lncRNAs CCAT1 and CCAT2 could be prognostic biomarkers in CRC [30]. These studies suggest that a comprehensive understanding of lncRNAs functions in CRC may help develop promising diagnostic and therapeutic strategies.

In this study, we identified aberrantly expressed lncRNAs in CRC by analyzing TCGA sequencing data and explored the functions of SNHG6 in CRC. We found that SNHG6 expression was upregulated in CRC tissues and cells, and its upregulation was induced by DNA copy number gains and SP1 activation. Besides, high SNHG6 expression indicated progression and poor prognosis of CRC. Functional experiments demonstrated that SNHG6 significantly promoted CRC growth and metastasis both in vitro and in vivo. In terms of mechanism, we found that SNHG6 could act as a molecular sponge of miR-26a, miR-26b, and miR-214 in the cytoplasm and exert its cancer-promoting effects by regulating EZH2, a common target of these microRNAs (Fig. 8h).

SNHG6 is located on chromosome 8q13, a genomic region frequently amplified in CRC. Guo et al. reported that SNHG6 could induce genome-wide hypomethylation by reducing the SAMe concentration by competitively binding miR-1297 in hepatocellular carcinoma (HCC) [16]. Chang et al. demonstrated that SNHG6 could regulate ZEB1 expression by sponging miR-101-3p and interacting with UPF1 in HCC [14]. Cao et al. reported that SNHG6 functioned as a competing endogenous RNA to promote HCC progression by regulating transforming growth factor-β-activated kinase 1 (TAK1) [15]. Interestingly, they also found that SNHG6 could act as a sponge of miR-26a/b. However, we found that SNHG6 could not regulate TAK1 expression in CRC cells (data not shown), and the discrepant results might be due to tissue-specific differences. Oncogenic roles of SNHG6 were also observed in gastric cancer, glioma, and osteosarcoma [12, 13, 17]. In addition, Li et al. revealed that high SNHG6 expression predicted poor prognosis in CRC [18]. However, they did not uncover how SNHG6 exerted its tumor-promoting effects.

PRC2 is an epigenetic regulator critical for multiple cellular processes, such as cell cycle regulation, cell apoptosis, and cell differentiation. Moreover, growing evidence indicates that PRC2 is involved in cancer initiation and progression [31]. EZH2 is the catalytic subunit of the PRC2, which catalyzes the trimethylation of lysine27 of histone3 and mediates the silencing of target genes. EZH2 is highly expressed in a wide range of cancer types, including CRC, and the overexpression of EZH2 is often correlated with advanced cancer stages and poor prognosis [20, 32, 33]. The upregulation of EZH2 expression is induced by a variety of factors. For example, c-Myc and STAT3 can bind to the promoter of EZH2 and directly activate its transcription. In addition to transcriptional regulators, multiple microRNAs, such as miR-26a, miR-26b, miR-101, miR-214, and let-7a, have been shown to directly regulate EZH2 expression. Many of these microRNAs are downregulated in cancers [19, 20, 34]. Besides, some lncRNAs, such as MALAT1, TP73-AS1, and ANCR, have been reported to regulate the expression and function of EZH2 [35–37]. In this study, we revealed that SNHG6 could regulate EZH2 expression by competitively binding miR-26a, miR-26b, and miR-214. Previous studies also reported that the expression levels of these microRNAs were all downregulated in CRC [38–42]. To date, many EZH2 targets have been identified. The INK4B-ARF-INK4A tumor suppressor locus is a well-known target for EZH2, and the suppression of these genes is important for cancer growth as well as embryo development [43, 44]. Another critical target of EZH2 in multiple cancers is the E-cadherin gene, whose downregulation is critical for EMT and metastasis [20, 45]. Here, we found that SNHG6 overexpression in CRC cells increased H3K27me3 enrichment in the promoters of P14^ARF^, P15^INK4b^, P16^INK4a^, and E-cadherin and downregulated their expression. These findings indicated that EZH2 and its targets were involved in oncogenic roles of SNHG6 in CRC.

## Conclusions

Our study revealed that SNHG6 expression was upregulated in CRC tissues and cells. High expression of SNHG6 was associated with tumor progression and poor survival. SNHG6 promoted CRC cell growth and metastasis by acting as a molecular sponge to regulate EZH2 and its targets. These data suggest that SNHG6 may be a promising biomarker and a novel therapeutic target of CRC.

## Additional files

Additional file 1: **Table S1.** The list of primers and probes. Table S2. SiRNAs and sh-RNAs sequence. Table S3. Information of antibodies. (DOCX 22 kb)

Additional file 2: Supplemental Materials and Methods. (DOCX 18 kb)

Additional file 3: **Figure S1.** High SNHG6 expression related to progression andpoor prognosis of CRC. Figure S2. SP1 is up-regulated in colorectal cancer and positively correlated with SNHG6. Figure S3. SNHG6 promotes CRC cell growth in vitro. Figure. S4 SNHG6 inhibits CRC cell apoptosis in vivo. Figure S5. MiR-214, miR-26a and miR-26b could bind to SNHG6 in CRC cells. Figure S6. SNHG6 promotes HCT-116 cells growth, migration and invasion by sponging miR-214, miR-26a and miR-26b. Figure S7. EZH2 is upregulated in CRC tissues and miR-214, miR-26a or miR-26b could inhibit its expression. (DOCX 7220 kb)

## Abbreviations

ceRNAs: Competing endogenous RNAs
ChIP: Chromatin immunoprecipitation assay
CRC: Colorectal cancer
DMEM: Dulbecco’s modified Eagle’s medium
EdU: Ethynyl deoxy Uridine
EMT: Epithelial-mesenchymal transition
ENCODE: Encyclopedia of DNA elements
EZH2: Enhancer of zeste homolog 2
FISH: RNA fluorescence in situ hybridization
GSEA: Gene set enrichment analysis
H3K27me3: Trimethylation of histone3 lysine27
HCC: Hepatocellular carcinoma
IF: Immunofluorescence
IHC: Immunohistochemistry
lncRNA: Long non-coding RNA
NC: Negative control
PRC2: Polycomb repressor complex 2
qRT-PCR: Quantitative reverse transcription polymerase chain reaction
RIP: RNA Immunoprecipitation assay
SD: Standard deviation
siRNAs: Small interfering RNAs
SNHG6: Small nucleolar RNA host gene 6
TAK1: Transforming growth factor-β-activated kinase 1
TCGA: The Cancer Genome Atlas
TUNEL: Terminal transferase dUTP nick end labeling
