# Supplementary material for: lncRNA SNHG6 regulates EZH2 expression by sponging miR-26a/b and miR-214 in colorectal cancer
Source: J Hematol Oncol. 2019 Jan 9;12:3. doi: 10.1186/s13045-018-0690-5 (PMC6327409; doi:10.1186/s13045-018-0690-5)
Supplement: Supplementary file 1 — Table S1. The list of primers and probes. Table S2. SiRNAs and sh-RNAs sequence. Table S3. Information of antibodies. (DOCX 22 kb) [file 13045_2018_690_MOESM1_ESM.docx]

| **Table S1： The list of primers and probes.** | |  |
| --- | --- | --- |
| **qPCR primers** |  |  |
|  | **Forward Primer** | **Reverse Primer** |
| SNHG6 | ATACTTCTGCTTCGTTACCT | CTCATTTTCATCATTTGCT |
| SP1 | TCCAGACCATTAACCTCAGTGC | TGTATTCCATCACCACCAGCC |
| EZH2 | AATCAGAGTACATGCGACTGAGA | GCTGTATCCTTCGCTGTTTCC |
| P16INK4 | ATGGAGCCTTCGGCTGACT | GTAACTATTCGGTGCGTTGGG |
| P14ARF | GGGTTTTCGTGGTTCACATCC | CTAGACGCTGGCTCCTCAGTA |
| CDH1 | ATTTTTCCCTCGACACCCGAT | TCCCAGGCGTAGACCAAGA |
| P15INK4b | CTGGACCTGGTGGCTACG | ACATTGGAGTGAACGCATCG |
| U6 | CTCGCTTCGGCAGCACA | AACGCTTCACGAATTTGCGT |
| GAPDH | GGGAGCCAAAAGGGTCATCA | TGATGGCATGGACTGTGGTC |
| **primers for copy number detection** | |  |
|  | **Forward Primer** | **Reverse Primer** |
| SNHG6-1 | CACAAGCCATTTGCGCAGTC | CCCTTGCAGGCACCTCCTTA |
| SNHG6-2 | TGCAAGAAAGCCTTTGAGGTGA | CAATACATGCCGCGTGATCC |
| SNHG6-3 | GAGGCTGACAGCTGGGGAGA | TTCAACCACCTGGTACAACAGCA |
| **primers for ChIP** | |  |
|  | **Forward Primer** | **Reverse Primer** |
| SNHG6 | CCACATAAACTTGCTGTGTGGC | CGACATCGGAAGACAGCTCTTAG |
| P14ARF | GTGGGTCCCAGTCTGCAGTTA | CCTTTGGCACCAGAGGTGAG |
| P15INK4b | TCTGGTAAGGGTGTGCTGTG | AAAACTCCTCTGTGGCATGTG |
| P16INK4a | ACCCCGATTCAATTTGGCAG | AAAAAGAAATCCGCCCCCG |
| E-cadherin | TAGAGGGTCACCGCGTCTAT | TCACAGGTGCTTTGCAGTTC |

| **Table S2: SiRNAs and sh-RNAs sequence.** | |  |
| --- | --- | --- |
| **siRNAs** |  |  |
|  | **sense sequence** | **anti-sense sequence** |
| si-SP1#1 | 5'-CCUGGAGUGAUGCCUAAUATT-3' | 5'-UAUUAGGCAUCACUCCAGGTT-3' |
| si-SP1#2 | 5'-GUGCAAACCAACAGAUUAUTT-3' | 5'-AUAAUCUGUUGGUUUGCACTT-3' |
| si-SNHG6#1 | 5'-GAAGAGCCCGUUAGUCAUGTT-3' | 5'-CAUGACUAACGGGCUCUUCTT-3' |
| si-SNHG6#2 | 5'-GAAGGUGUAUGAAAGUCAUTT-3' | 5'-AUGACUUUCAUACACCUUCTT-3' |
| si-EZH2 | 5'-CGGCUUCCCAAUAACAGUATT-3' | 5'-UACUGUUAUUGGGAAGCCGTT-3' |
| **shRNAs** | **target sequence** |  |
| sh-SNHG1#1 | CGCGAAGAGCCGTTAGTCATGCCGGTGTG | |

| **Table S3: Information of antibodies.** | | |  |  |  |  |
| --- | --- | --- | --- | --- | --- | --- |
|  |  | **Western blot** | **ChIP** | **RIP** | **IHC** | **IF** |
| SP1(D4C3) Rabbit mAb | cell signaling technology #9389 |  | 1:100 |  | 1:200 |  |
| Anti-p15 INK4b | abcam #ab53034 | 1:1000 |  |  | 1:100 |  |
| Anti-CDKN2A/p14ARF | abcam #ab185620 | 1:1000 |  |  |  |  |
| Anti-CDKN2A/p16INK4a | abcam #ab108349 | 1:1000 |  |  |  |  |
| Tri-Methyl-Histone H3 (Lys27) (C36B11) Rabbit mAb | cell signaling technology #9733 |  | 1:100 |  |  |  |
| Ezh2 (D2C9) XP Rabbit mAb | cell signaling technology #5246 | 1:1000 |  |  | 1:50 |  |
| CDK4 (D9G3E) Rabbit mAb | cell signaling technology #12790 | 1:1000 |  |  |  |  |
| CDK6 (D4S8S) Rabbit mAb | cell signaling technology #13331 | 1:1000 |  |  |  |  |
| Cyclin D1 Mouse Monoclonal antibody | proteintech #60186-1-lg | 1:2000 |  |  |  |  |
| Bax (D2E11) Rabbit mAb | cell signaling technology #5023 | 1:1000 |  |  |  |  |
| Caspase-3 Antibody | abcam #ab9662 | 1:1000 |  |  |  |  |
| PARP (46D11) Rabbit mAb | cell signaling technology #9532 | 1:1000 |  |  |  |  |
| Argonaute 2 (C34C6) Rabbit mAb | cell signaling technology #2897 | 1:1000 |  | 1:100 |  |  |
| KI67 Rabbit Polyclonal antibody | proteintech #27309-1-AP |  |  |  | 1:4000 |  |
| GAPDH (D16H11) XP Rabbit mAb | cell signaling technology #5174 | 1:1000 |  |  |  |  |
| Vimentin Rabbit Polyclonal | proteintech #10366-1-AP | 1:1000 |  |  | 1:1000 | 1:50 |
| E-cadherin Rabbit Polyclonal | proteintech #20874-1-AP | 1:1000 |  |  | 1:500 | 1:20 |
| MMP-9 (D6O3H) Rabbit mAb | cell signaling technology #13667 | 1:1000 |  |  |  |  |
