# Supplementary material for: lncRNA SNHG6 regulates EZH2 expression by sponging miR-26a/b and miR-214 in colorectal cancer
Source: J Hematol Oncol. 2019 Jan 9;12:3. doi: 10.1186/s13045-018-0690-5 (PMC6327409; doi:10.1186/s13045-018-0690-5)
Supplement: Supplementary file 2 — Supplemental Materials and Methods. (DOCX 18 kb) [file 13045_2018_690_MOESM2_ESM.docx]

**Supplemental Materials and Methods**

**Cell growth and colony formation assays**

For CCK-8 assay, HCT-116 and HCT-8 cells were seeded into 96-well plates at the density of 1×10^3^ (cells/well), and the absorbance at 450nm was measured on days 1, 2, 3, 4 and 5 with 10 μl of CCK-8 solution treated. For cell colony formation assays, 24 hours after transfection, 500 HCT-116 or HCT-8 cells were incubated in 6-well plates at 37˚C, 5% CO_2_. Two weeks later, the cells were stained with crystal violet (0.2%) for 30 minutes and the colony numbers were counted.

**5-Ethynyl-20-deoxyuridine (EdU) incorporation assay**

HCT-116 and HCT-8 cells were seeded at a density of 5×10^3^ cells per well in 96-well plates and cultured overnight. The newly synthesized DNA of the cells was assessed by the EdU incorporation assay using a Cell-Light EdU DNA Cell Proliferation Kit (Ribobio, China), according to the manufacturer’s instructions. The EdU incorporation rate was expressed as the ratio of EdU positive cells (red cells) to total Hoechst33342 positive cells (blue cells).

**Flow cytometry**

For cell cycle analysis, HCT-116 and HCT-8 cells were firstly trypsinized and washed with cold PBS, then they were fixed in 70% ethanol for 24 hours and stained with Propidium Iodide (PI) for 30 minutes. Finally, the cells were analyzed by FACScan flow cytometer (BD, USA). The cell cycle data was analyzed by ModFit LT software (Veirty, USA). Cell apoptosis was analyzed using the Annexin V-FITC/ (PI) Apoptosis Detection Kit (BD, USA) according to the protocol. Cells were stained with FITC and PI and then analyzed using FACScan (BD, USA). The cell apoptosis data were analyzed by Flowjo software (Tree Star, USA).

**Protein extraction and western blot**

Total proteins were extracted from cultured cells using cell lysis buffer. Then, the protein samples were loaded onto 10% sodium dodecyl sulfate polyacrylamide gel electrophoresis. The membranes were blocked with 5 % non-fat milk in Tris-buffered saline and incubated with a specific primary antibody and a secondary antibody. Protein expression was detected by enhanced chemiluminescence kit. Antibodies used in this study were listed in Table S2 (Additional file 2).

**Immunohistochemistry (IHC) and immunofluorescence (IF)**

IHC staining was performed using Dako Envision System (Dako, USA) according to the manufacturer’s guidelines. The IHC-stained tissue sections were scored by two pathologists who were blinded to the clinical parameters, respectively. The percentage of immunostaining and the staining intensity (0, negative; 1+, weak; 2+, moderate; and 3+, strong) were recorded. An H-score was calculated using the following formula: [1 × (% cells 1+) + 2 × (% cells 2+) + 3 × (% cells 3+)] × 100. The maximum H-score would be 300, corresponding to 100% of cells with strong intensity. For IF, cells were fixed in 4% paraformaldehyde, permeabilized using 0.5% Triton X-100 and incubated with primary antibody and secondary antibodies according to the manufacturer’s protocol. Coverslips were counterstained with DAPI and imaged with a confocal laser scanning microscope (Olympus FV1000). Antibodies information was listed in Table S2 (Additional file 2).

**Wound healing assay**

HCT-116 and HCT-8 cells (1×10^6^ cells/well) were treated with the indicated reagents, and wounds were made using a 100-μl plastic pipette tip. After making wounds, those cells were cultured with serum-free medium. The size of the wound was measured after 36 hours after wound formation, and the wound was imaged. The cell migration area was measured between dashed regions using ImageJ software (Bethesda, USA) and normalized to control cells.

**Transwell migration and matrigel invasion assays**

The migration and matrigel invasion assays were conducted using transwell chambers, which were coated with (invasion assay) or without (migration assay) the matrigel mix (BD, USA) according to the manufacturer’s protocol. The homogeneous serum-free single cell suspensions (1 **×** 10^5^ cells**/**well for migration and 5 **×** 10^5^**/**well for invasion, respectively) were added to the upper chambers and medium with 10% fetal bovine serum was added into the lower chambers, then incubated for 24 hours. The cells that had migrated or invaded through the membrane to the lower surface were fixed, stained and counted.

**TUNEL assay**

The TUNEL assay was used to detect the apoptotic cells in tissues with the Cell Death Detection Kit (Roche, USA). Brieﬂy, paraffin-embedded slides were deparaffinized and rehydrated. Then, they were permeabilized in 0.1% Triton X-100 and then incubated with the TUNEL reaction mixture for 1 hour at 37°C. After the slides were washed with PBS, they were incubated with peroxidase-conjugated antibody for 30 min at 37°C and were developed with the DAB system. The apoptosis index was calculated by counting the number of brown-stained nuclei, and the total number of cells measured by light microscopy represented at least 10 fields submitted to investigator-blinded counting. The results are expressed as a percentage of the number of apoptotic cells/total cells.
