# Supplementary material for: lncRNA SNHG6 regulates EZH2 expression by sponging miR-26a/b and miR-214 in colorectal cancer
Source: J Hematol Oncol. 2019 Jan 9;12:3. doi: 10.1186/s13045-018-0690-5 (PMC6327409; doi:10.1186/s13045-018-0690-5)
Supplement: Supplementary file 3 — Figure S1. High SNHG6 expression related to progression andpoor prognosis of CRC. Figure S2. SP1 is up-regulated in colorectal cancer and positively correlated with SNHG6. Figure S3. SNHG6 promotes CRC cell growth in vitro. Figure. S4 SNHG6 inhibits CRC cell apoptosis in vivo. Figure S5. MiR-214, miR-26a and miR-26b could bind to SNHG6 in CRC cells. Figure S6. SNHG6 promotes HCT-116 cells growth, migration and invasion by sponging miR-214, miR-26a and miR-26b. Figure S7. EZH2 is upregulated in CRC tissues and miR-214, miR-26a or miR-26b could inhibit its expression. (DOCX 7220 kb) [file 13045_2018_690_MOESM3_ESM.docx]

**Figure S1:**

**
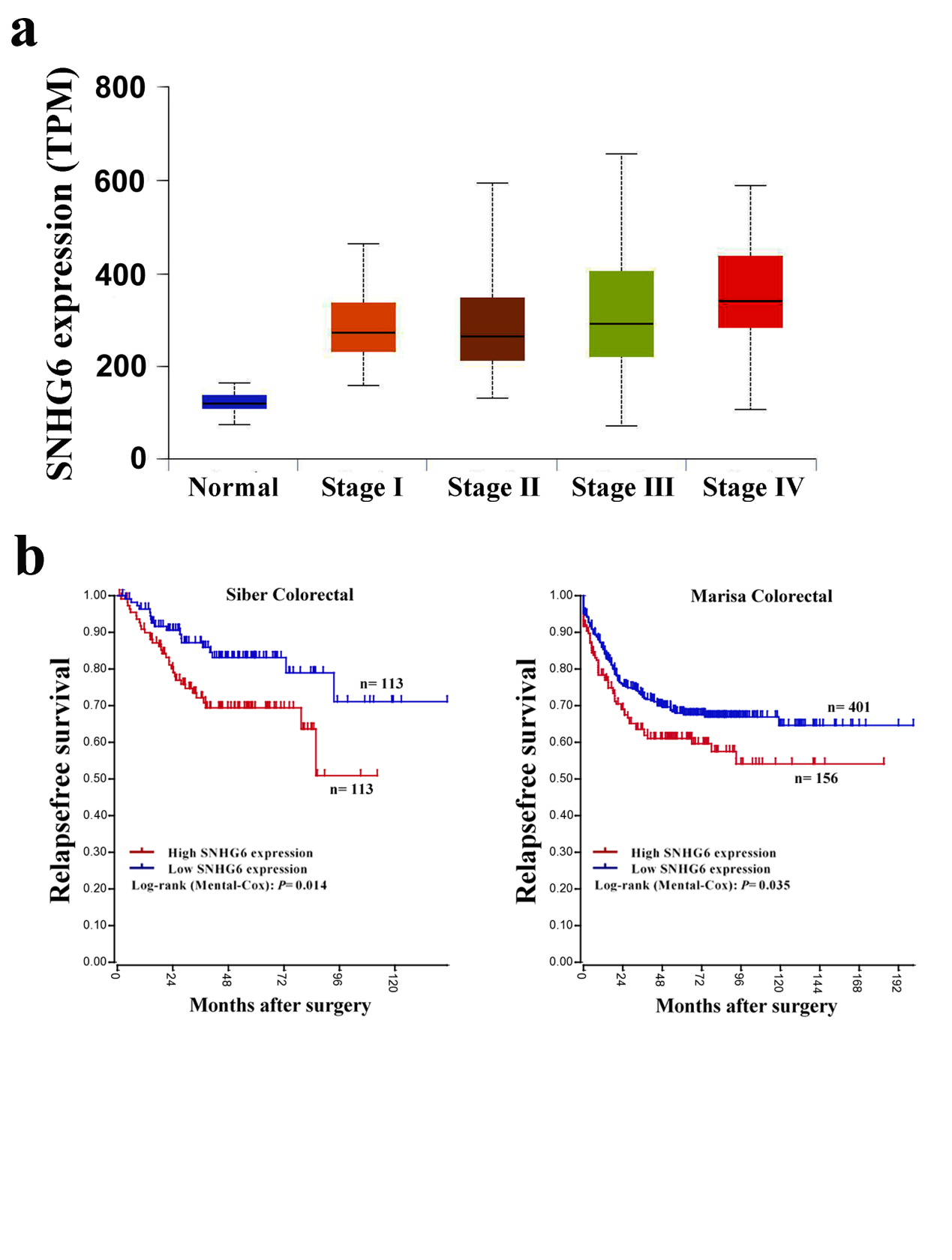
**

**Figure. S1** High SNHG6 expression related to progression and poor prognosis of CRC. **a** The SNHG6 expression in different tumor stages of CRC in TCGA cohort. **b** Kaplan–Meier survival analysis showed that high SNHG6 expression was significantly correlated with poor disease-free survival in two independent CRC cohort in R2 database.

**Figure S2:**


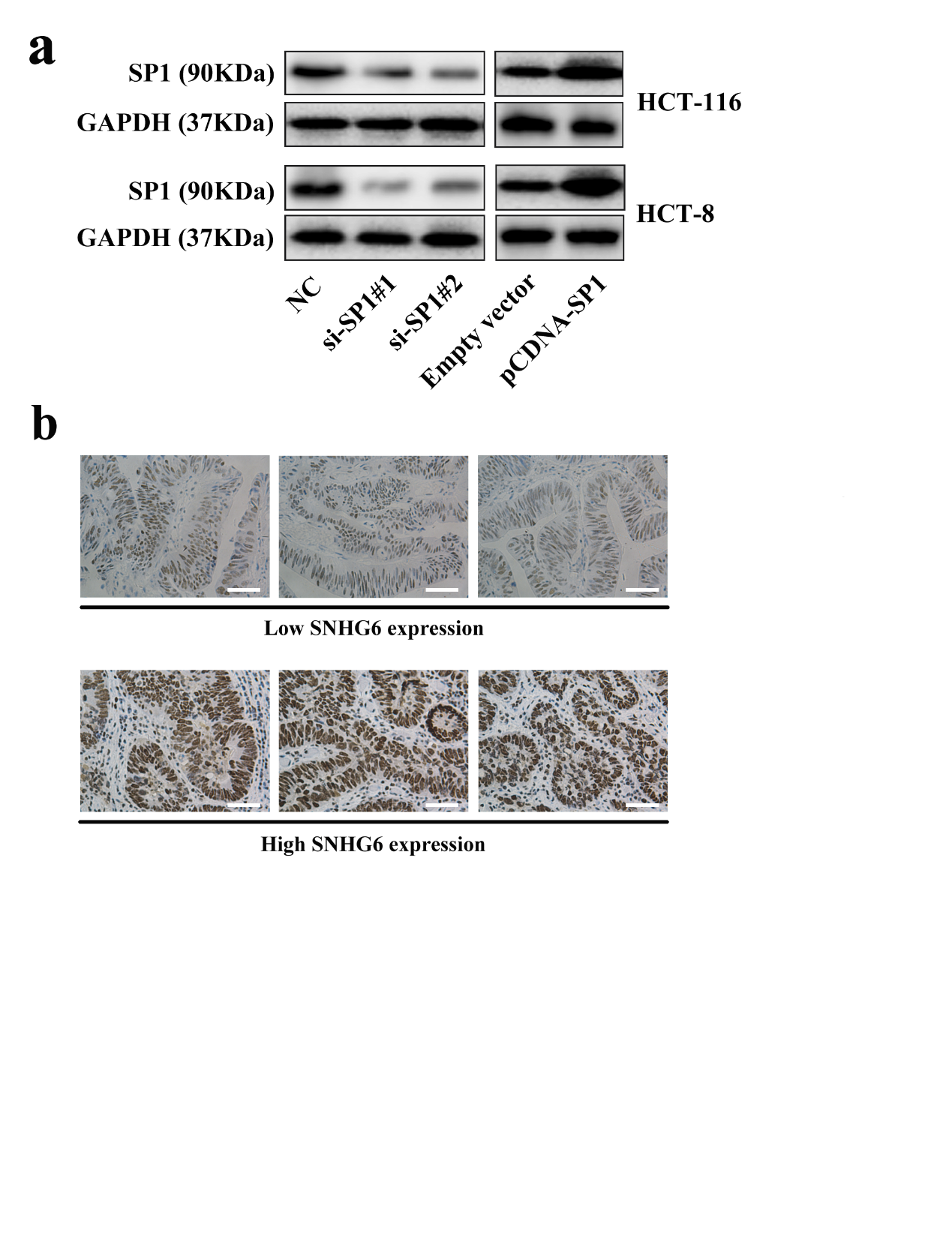


**Figure. S2** SP1 is up-regulated in colorectal cancer and positively correlated with SNHG6. **a** SP1 expression were detected by western blotting in SP1 knockdown and overexpressed CRC cells. **b** SP1 was elevated in SNHG6 relatively high expressed CRC tissues. Scare bar = 50μm.

**Figure S3:**


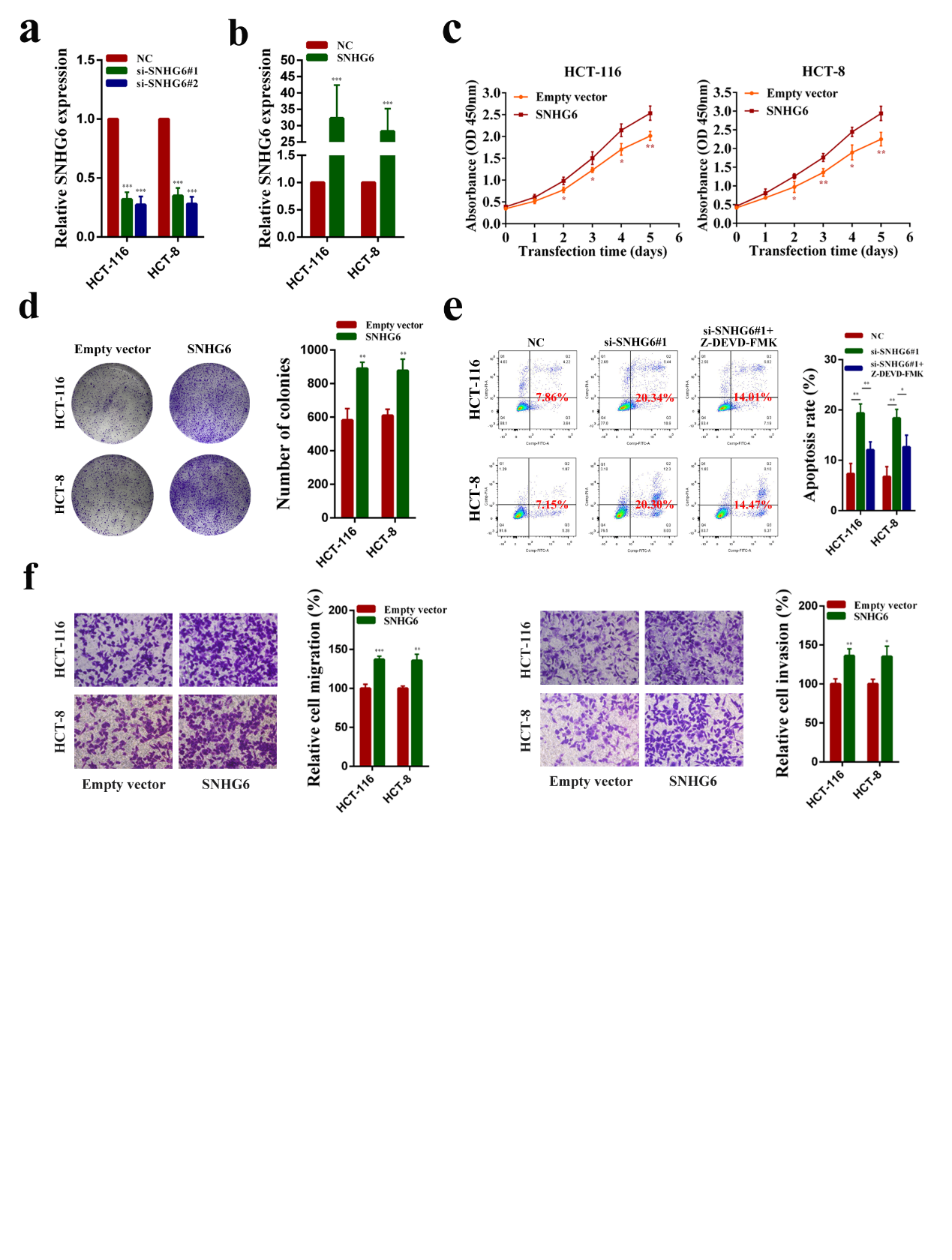


**Figure. S3** SNHG6 promotes CRC cell growth *in vitro*. **a** SNHG6 were quantified by qRT-PCR after transfection of SNHG6 siRNAs in CRC cells. **b** SNHG6 were quantified by qRT-PCR after transfection of SNHG6 overexpression vectors in CRC cells. **c** HCT-116 and HCT-8 cells transfected with the SNHG6 overexpression vectors were subjected to the CCK-8 assay. **d** HCT-116 and HCT-8 cells transfected with SNHG6 overexpression vectors were seeded into 6-well plates. The number of colonies was counted on the 14th day after seeding. **e** The effect of Caspase-3 inhibitor Z-DEVD-FMK on SNHG6 knockdown CRC cells was analyzed by flow cytometric cell apoptosis assays. **f** Transwell assays were used to determine the invasion and migration abilities of CRC cells transfected with the SNHG6 overexpression vector. *P < 0.05, **P < 0.01 and ***P < 0.001.

**Figure S4:**

**
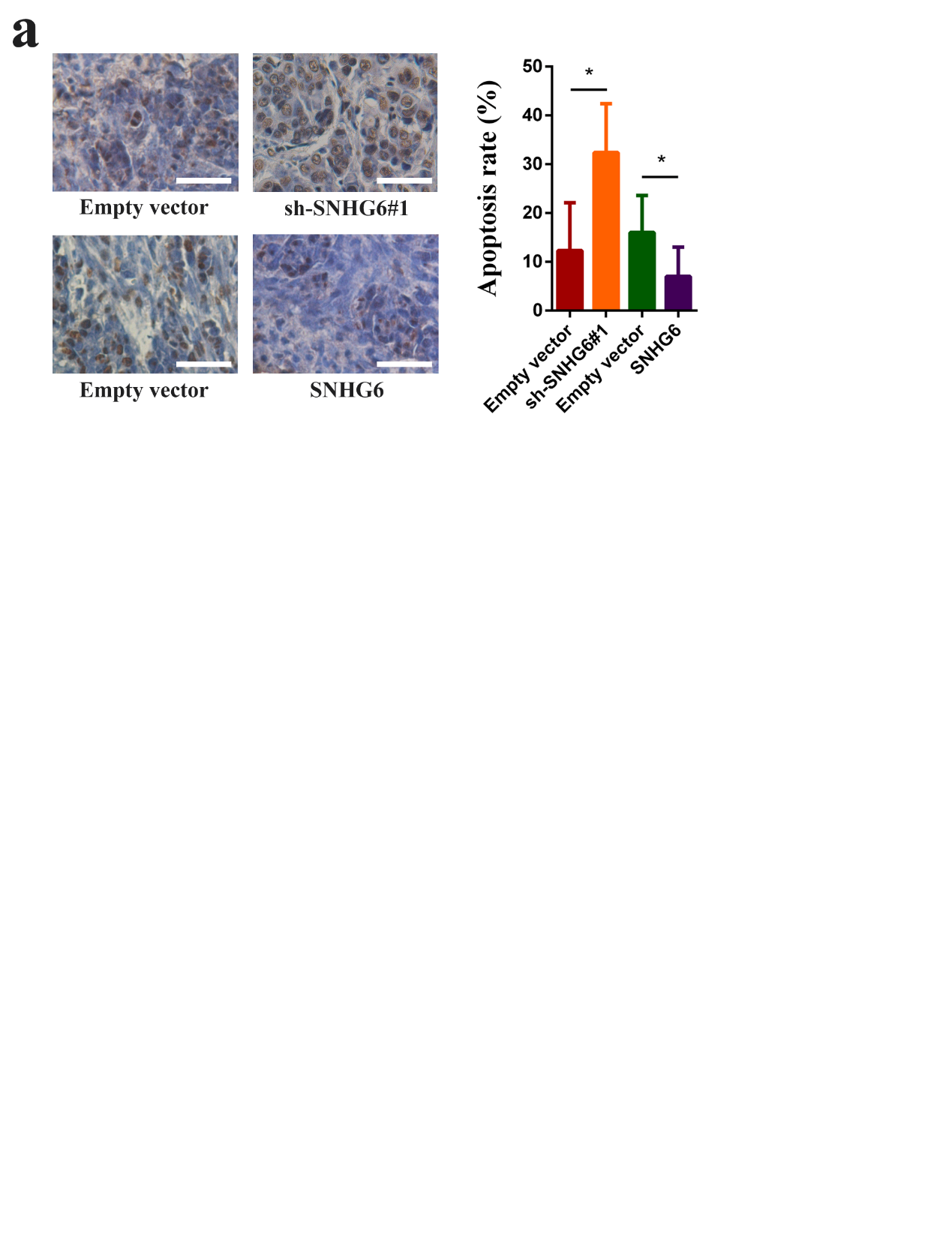
**

**Figure. S4** SNHG6 inhibits CRC cell apoptosis *in vivo*. **a** Left panel, representative images of the TUNEL assays of tumor samples from the different groups. Right panel, the statistical result of the TUNEL assays. Scare bar = 50 μm. *P < 0.05.

**Figure S5:**


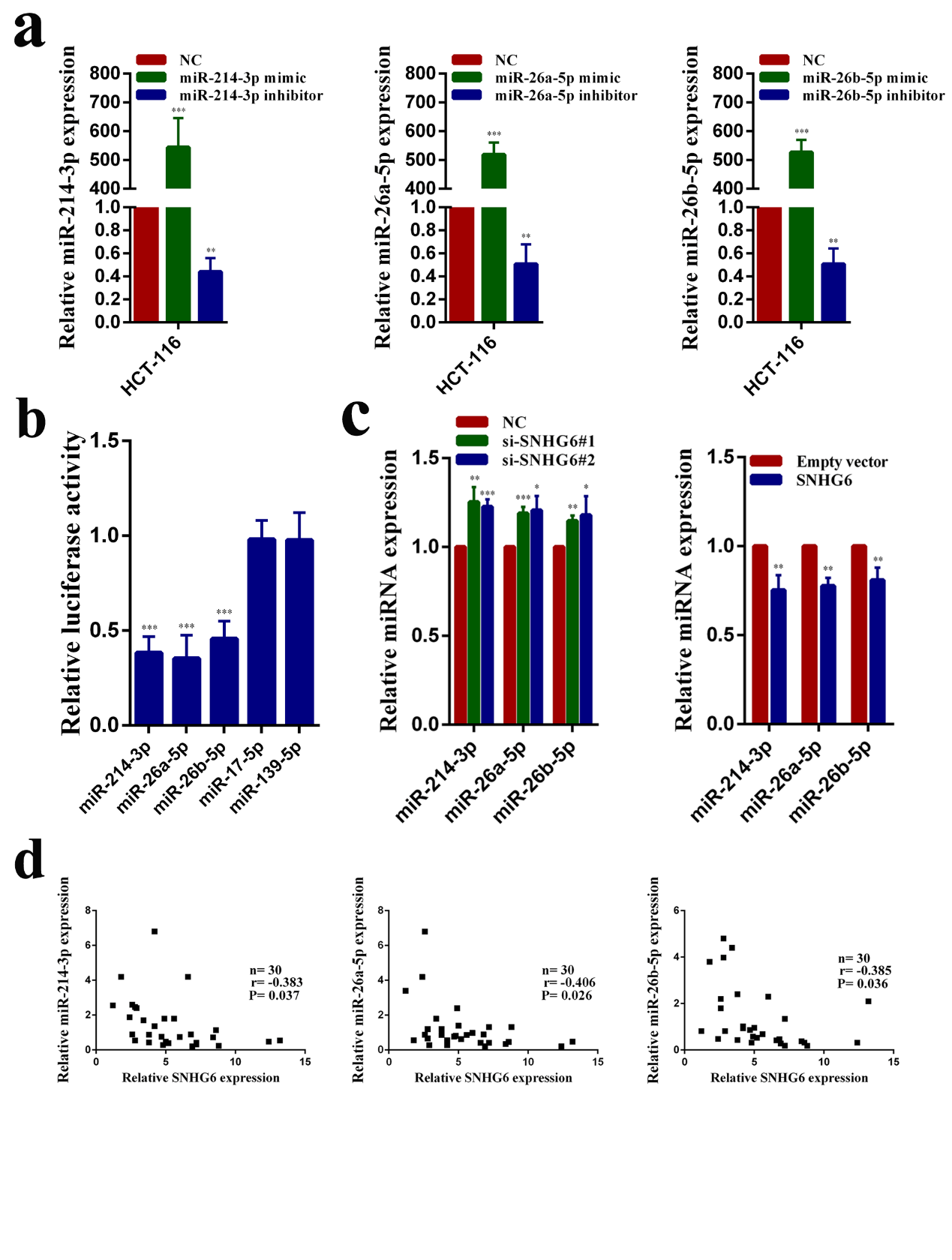


**Figure. S5** MiR-214, miR-26a and miR-26b could bind to SNHG6 in CRC cells. **a** MiR-214, miR-26a and miR-26b were detected by qRT-PCR after transfection of their mimics or inhibitors. **b** We constructed corresponding dual luciferase reporter vector according to predicted binding sites in SNHG6 for these microRNA. Results of luciferase reporter assays were presented as ratio of luciferase activity of transfection with mimics to luciferase activity transfection with NC. **c** MiR-214, miR-26a and miR-26b were detected by qRT-PCR after transfection of SNHG6 siRNAs or overexpression vectors in HCT-116 cells. **d** The correlation between SNHG6 and miR-214, miR-26a or miR-26b expression analyzed in 30 paired CRC samples. **P* < 0.05, ***P <* 0.01 and ****P <* 0.001.

**Figure S6:**


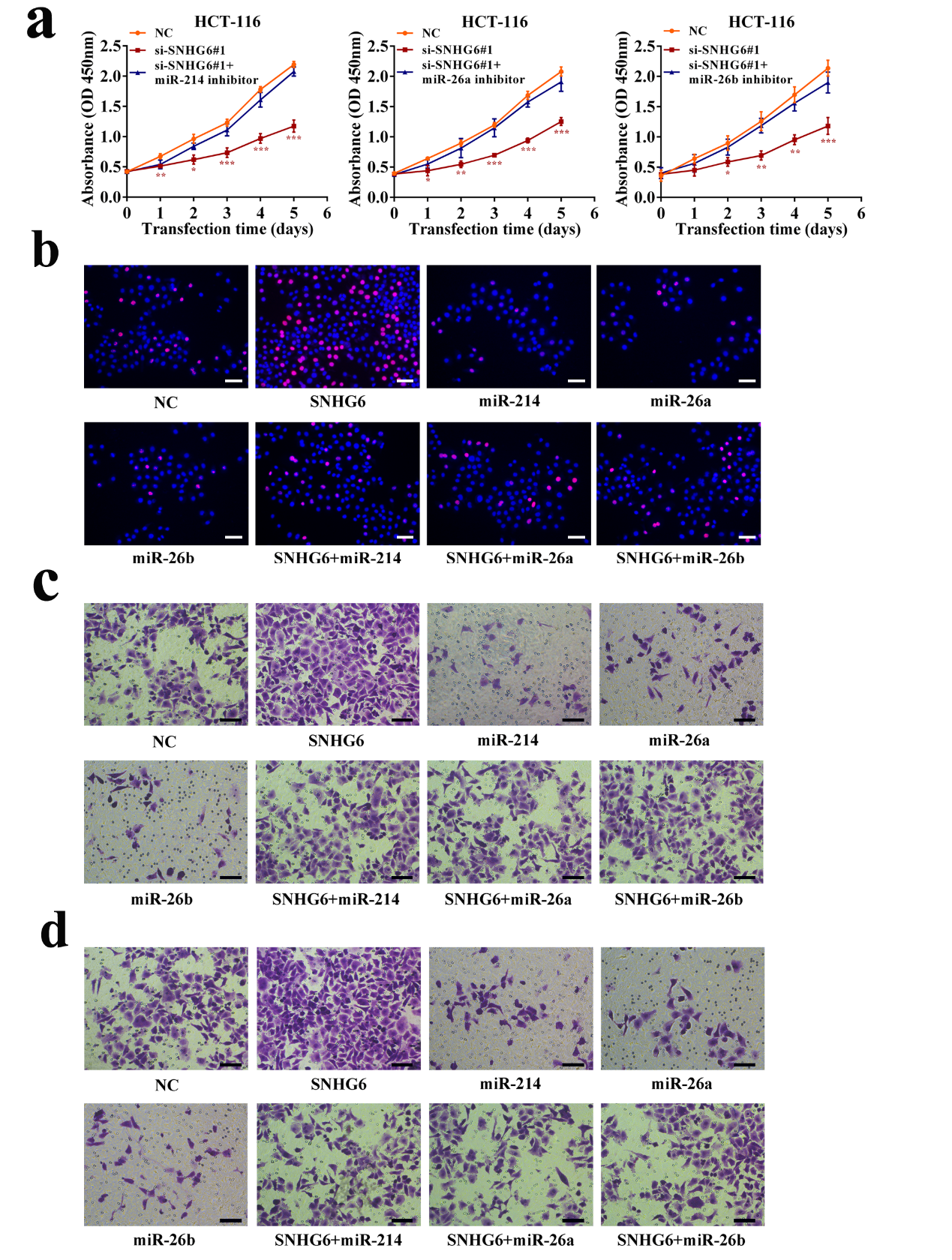


**Figure. S6** SNHG6 promotes HCT-116 cells growth, migration and invasion by sponging miR-214, miR-26a and miR-26b. **a** CCK-8 assays of SNHG6 knockdown HCT-116 cells with transfection of miR-214, miR-26a or miR-26b inhibitors. **b** Representative images of EdU assays performed using HCT-6 cells with indicated treatment. **c** and **d** Representative images of transwell migration or invasion assays performed using HCT-6 cells with indicated treatment. Scare bar = 50μm. **P* < 0.05, ***P <* 0.01 and ****P <* 0.001.

**Figure S7:**


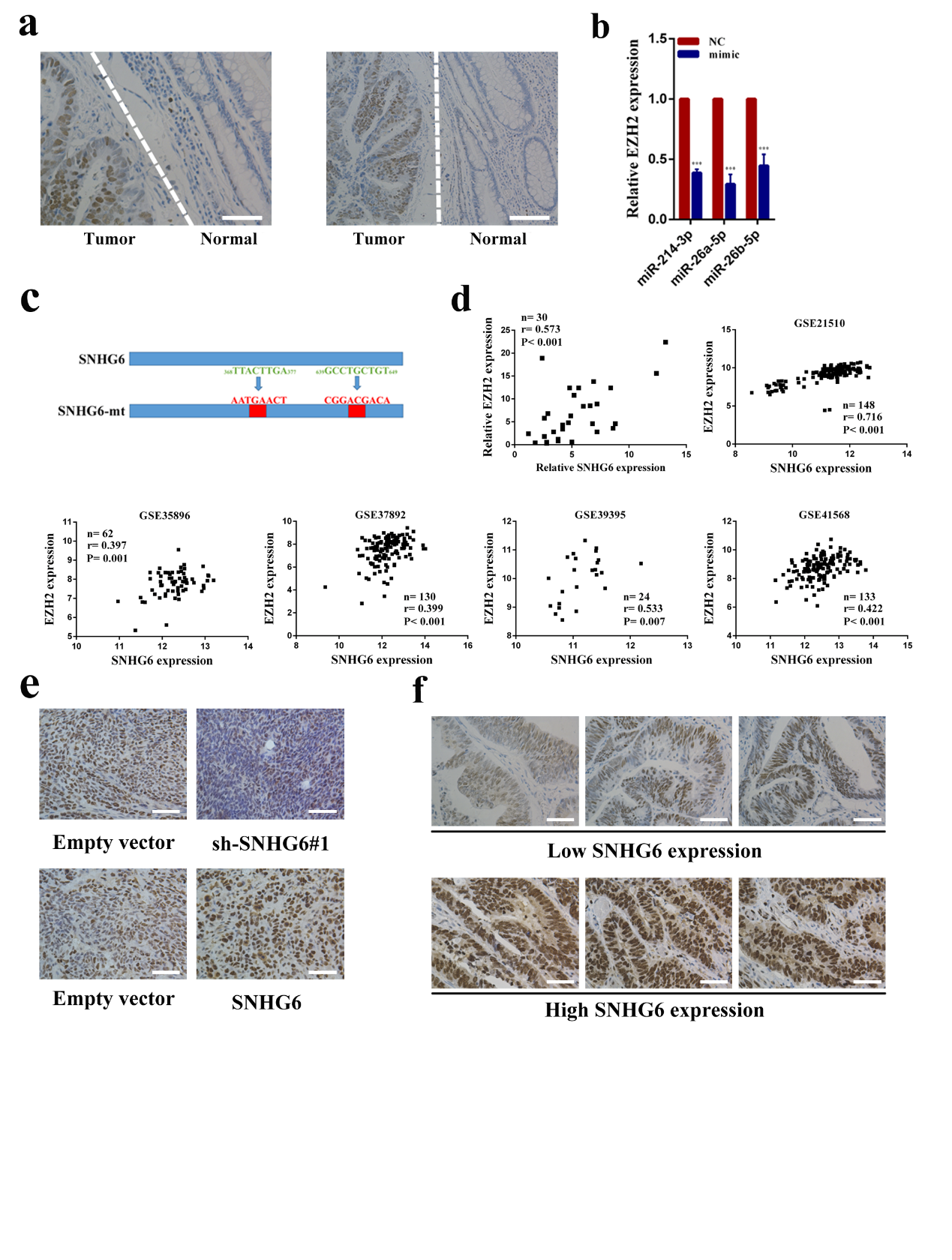


**Figure. S7** EZH2 is upregulated in CRC tissues and miR-214, miR-26a or miR-26b could inhibit its expression. **a** EZH2 protein levels in CRC tissues and corresponding normal tissues was detected by IHC. **b** Detection of EZH2 in HCT-116 cells by qRT-PCR after transfection of miR-214, miR-26a or miR-26b mimics. **c** Schematic outline of mutated sites (predicted microRNA binding sites) on SNHG6-mt. **d** Pearson correlation analysis between SNHG6 and EZH2 levels in CRC tissues (Our cohort and five independent cohorts). **e** EZH2 expression was detected by IHC xenograft tumor tissues. **f** EZH2 expression was detected by IHC in CRC tissues with relatively low or high SNHG6 expression. Scare bar = 50μm. ****P <* 0.001.
